# The Influence of Extracerebral Tissue on Continuous Wave Near-Infrared Spectroscopy in Adults: A Systematic Review of In Vivo Studies

**DOI:** 10.3390/jcm12082776

**Published:** 2023-04-08

**Authors:** Nick Eleveld, Diana C. Esquivel-Franco, Gea Drost, Anthony R. Absalom, Clark J. Zeebregts, Jean-Paul P. M. de Vries, Jan Willem J. Elting, Natasha M. Maurits

**Affiliations:** 1 Department of Neurology, University Medical Centre Groningen, University of Groningen, Postbus 30001, 9700 RB Groningen, The Netherlands; 2 Department of Neurosurgery, University Medical Centre Groningen, University of Groningen, Postbus 30001, 9700 RB Groningen, The Netherlands; 3 Department of Anaesthesiology, University Medical Centre Groningen, University of Groningen, Postbus 30001, 9700 RB Groningen, The Netherlands; 4 Department of Surgery, Division of Vascular Surgery, University Medical Centre Groningen, University of Groningen, Postbus 30001, 9700 RB Groningen, The Netherlands

**Keywords:** near-infrared spectroscopy, extracerebral tissue, extracranial tissue, cerebral oxygen saturation, adult population, systematic review

## Abstract

Near-infrared spectroscopy (NIRS) is a non-invasive technique for measuring regional tissue haemoglobin (Hb) concentrations and oxygen saturation (rSO_2_). It may be used to monitor cerebral perfusion and oxygenation in patients at risk of cerebral ischemia or hypoxia, for example, during cardiothoracic or carotid surgery. However, extracerebral tissue (mainly scalp and skull tissue) influences NIRS measurements, and the extent of this influence is not clear. Thus, before more widespread use of NIRS as an intraoperative monitoring modality is warranted, this issue needs to be better understood. We therefore conducted a systematic review of published in vivo studies of the influence of extracerebral tissue on NIRS measurements in the adult population. Studies that used reference techniques for the perfusion of the intra- and extracerebral tissues or that selectively altered the intra- or extracerebral perfusion were included. Thirty-four articles met the inclusion criteria and were of sufficient quality. In 14 articles, Hb concentrations were compared directly with measurements from reference techniques, using correlation coefficients. When the intracerebral perfusion was altered, the correlations between Hb concentrations and intracerebral reference technique measurements ranged between |r| = 0.45–0.88. When the extracerebral perfusion was altered, correlations between Hb concentrations and extracerebral reference technique measurements ranged between |r| = 0.22–0.93. In studies without selective perfusion modification, correlations of Hb with intra- and extracerebral reference technique measurements were generally lower (|r| < 0.52). Five articles studied rSO_2_. There were varying correlations of rSO_2_ with both intra- and extracerebral reference technique measurements (intracerebral: |r| = 0.18–0.77, extracerebral: |r| = 0.13–0.81). Regarding study quality, details on the domains, participant selection and flow and timing were often unclear. We conclude that extracerebral tissue indeed influences NIRS measurements, although the evidence (i.e., correlation) for this influence varies considerably across the assessed studies. These results are strongly affected by the study protocols and analysis techniques used. Studies employing multiple protocols and reference techniques for both intra- and extracerebral tissues are therefore needed. To quantitatively compare NIRS with intra- and extracerebral reference techniques, we recommend applying a complete regression analysis. The current uncertainty regarding the influence of extracerebral tissue remains a hurdle in the clinical implementation of NIRS for intraoperative monitoring. The protocol was pre-registered in PROSPERO (CRD42020199053).

## 1. Introduction

Cerebral near-infrared spectroscopy (NIRS) is a non-invasive technique used to measure and monitor tissue haemoglobin (Hb) concentrations and the oxygen saturation (rSO_2_) of the brain [1,2,3,4]. It is a promising technique for monitoring adequacy of cerebral perfusion and tissue oxygenation in patients that are at risk of peri-operative cerebral ischemia or hypoxia, such as those undergoing cardiothoracic or carotid surgery [3,5].

For cerebral NIRS, a light source on the scalp is used to emit light in the near-infrared (NIR) frequency range into the head. Only a small percentage of the emitted light is received by a detector on the scalp a few centimetres away because of the scattering and reflection of NIR light by human (brain) tissue. Light of specific frequencies can also be absorbed if there are sufficient concentrations of chromophores along the light path. Oxygenated (OxyHb) and deoxygenated haemoglobin (HHb) are both chromophores, but they have distinct NIR light absorption profiles. By using light of multiple wavelengths, and application of the Beer–Lambert law, which relates attenuation of light to the properties of the material through which it is travelling, OxyHb and HHb concentrations can be estimated. This allows us to calculate the regional tissue oxygen saturation (rSO_2_) as the ratio of OxyHb to total haemoglobin (tHb, sum of OxyHb and HHb) [6].

In patients undergoing procedures that pose a risk to cerebral perfusion, changes in cerebral tissue oxygenation are possible while systemic oxygenation is unchanged. As clinicians may use NIRS-based measurements during surgical procedures to inform clinical management decisions, it is important to know to what extent the obtained measurements reflect purely cerebral physiology [6,7].

When NIRS technology is used for cerebral measurements, an important challenge is that the light reaching the detector optode has also passed through extracerebral tissues. These tissues, which include scalp, skull, dura mater, and cerebrospinal fluid, can all influence the amount of light received by the detector, as they can reflect and scatter the light. They also contain molecules such as haemoglobin and myoglobin that can absorb NIR light [8,9,10,11]. Simulation studies suggest that extracerebral tissues have a large influence on NIRS measurements [9]. In these studies, the results varied according to the location on the head and source–detector separation (SDS); however, of the total amount of emitted light that was absorbed prior to reaching the detector optode, only 6 to 20% had been absorbed by the cortical grey matter. Most of the attenuation or absorption occurred during passage through the scalp and skull. However, these studies used computer simulations that assume certain anatomical and optical properties of the head tissues, which may not always be valid in vivo [9].

Despite these disadvantages, cerebral NIRS has continued to be used extensively in scientific and clinical practice [3,12,13]. To better extract the intracerebral (grey and white matter) NIRS components, manufacturers have developed NIRS devices with multiple sources and detectors and digital filtering techniques [4]. It could therefore be that the influence of extracerebral tissue is less pronounced in practice than in theory.

Surprisingly, even though NIRS devices have been used for decades, there is no consensus on the degree to which extracerebral tissue influences cerebral NIRS measurements. Moreover, there is currently no unified method to determine how these tissue layers influence NIRS measurements. Thus, before more widespread use of NIRS as an intraoperative monitoring modality is warranted, these issues need to be better understood. We therefore performed a systematic review of the literature, assessed several study protocols, determined methodological quality, and extracted quantitative results. We discuss the current methodological challenges and provide practical recommendations.

## 2. Materials and Methods

A systematic review was conducted following the Preferred Reporting Items for Systematic Reviews and Meta-Analysis of Diagnostic Test Accuracy Studies (PRISMA-DTA) Statement [14]. PRISMA-DTA checklists are provided as supplementary materials. The protocol was registered in the International Prospective Register of Systematic Reviews (PROSPERO) database, under registration number CRD42020199053.

### 2.1. Research Question

The research question was designed following the Population, Exposure, Outcome (PEO) format: ‘What is the influence of extracerebral tissue (Exposure) on cerebral near-infrared spectroscopy (Outcome) in adult humans (Population)?’

### 2.2. Search Strategy

A broad search strategy was used to generate a comprehensive overview of all published literature investigating the influence of extracerebral tissue on NIRS. Specifically, clinical and non-clinical human adult studies, and pre-clinical studies involving large adult animals as a model for humans, were included. Four research databases were used: Scopus, Embase, Web of Science, and PubMed.

### 2.3. Search Concepts

Two search concepts were used to screen potentially relevant studies: ‘extracerebral tissue’ and ‘near-infrared spectroscopy’. Titles, abstracts, keywords, and (if applicable) Medical Subject Headings (MESH) or Emtree terms were included in the search strategy. The search was conducted on 2 February 2022. No restriction on the year of publication was applied. The complete literature search strategy can be found in File S1. The references of included articles were screened for overlooked articles.

### 2.4. Inclusion Criteria

Eligibility was determined based on three elements corresponding to the PEO research question and two elements regarding study quality and methodology, as described in detail in Table 1. Population: our scope was limited to the adult population, since adults have a thicker scalp and skull than children [15,16], making NIRS measurements in adults more susceptible to interference by extracerebral tissue. Animal studies that can be considered a reasonable model for adult human cerebral NIRS were also included, as these studies often employ comprehensive, invasive monitoring techniques and study protocols. Outcome measure: we limited our scope to continuous wave and frequency-domain NIRS devices, as they are most commonly used in research and clinical practice. Exposure: we only included studies that specifically investigated the influence of intracerebral or extracerebral tissue layers on NIRS measurements. Studies that investigate the influence of systemic hemodynamic changes, for example, during functional NIRS studies, are therefore not included in the current review. Moreover, the included studies had to use reference techniques that measure blood flow, Hb concentrations or the tissue oxygen saturation of both the intra- and extracerebral tissue, or they had to apply a protocol in which the intra- or extracerebral tissue perfusion was selectively altered.

Articles were screened on title and abstract first, and subsequently on full text. Two researchers (NE, DE) screened the articles, and a consistency check was performed on 100 articles. For all articles, uncertainty regarding any inclusion criterion was discussed in a consensus meeting until agreement was reached.

### 2.5. Critical Appraisal

The Quality Assessment of Diagnostic Accuracy Studies 2 (QUADAS-2) tool was used to appraise the quality of included studies [17]. It was extended with the critical appraisal domain ‘Perfusion Modification Protocol’, and some questions were modified to fit the scope of our review. Details of the modified QUADAS-2 criteria are provided in Table S1.

### 2.6. Software and Statistical Analysis

Screening, data extraction, and critical appraisal were performed by two authors (NE, DE) in CADIMA [18]. Quantitative synthesis was performed in MATLAB (MATLAB (R2019b), The MathWorks Inc., Natick, MA, USA). The code can be found in File S2. Correlation figures were created using a Python 3.4 script adapted from that used by Bernaldo de Quiros and co-workers [19]. If the required data were only represented graphically in an article, the quantitative results were obtained from the graph using Webplotdigitizer [20].

If an article reported results from different NIRS devices, source–detector configurations, NIRS indices (Hb indices or rSO_2_), or they used different experimental protocols; these were identified as individual sub-studies and analysed in this review as such.

Group characteristics were expressed by median and inter-quartile range (IQR). Relations between NIRS and reference technique measurements were expressed as Pearson’s or Spearman’s correlation (‘r’), if available. If correlations were Fisher Z-transformed, these were transformed back.

## 3. Results

### 3.1. Literature Screening

**Figure 1:**
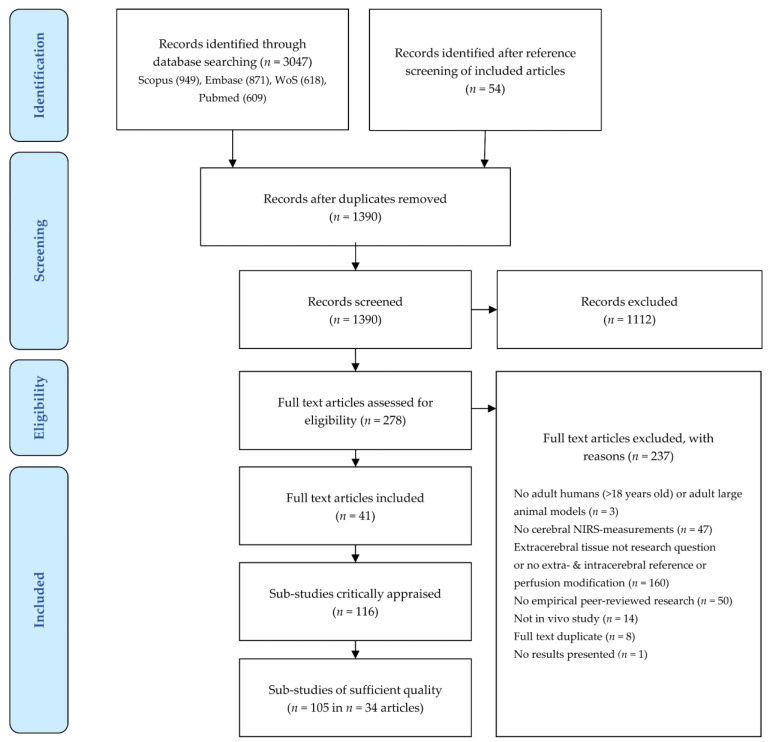
Screening process. Screening followed the Preferred Reporting Items for Systematic Reviews and Meta-Analysis of Diagnostic Test Accuracy Studies (PRISMA-DTA) Statement [14]. Exclusion of full text articles was possible based on multiple criteria. Sub-studies of sufficient quality are those for which more than 50% of the QUADAS-2 domains are assessed as ‘Low Risk of Bias’. NIRS, near-infrared spectroscopy; QUADAS-2, Quality Assessment of Diagnostic Accuracy Studies, revised second version; WoS, Web of Science. Figure adapted from Moher and colleagues, with permission [22].

An overview of the screening process is provided in Figure 1. The initial search yielded 3047 articles. A further 54 potential articles were identified by screening the references of the included articles. The interrater agreement was 84%, κ = 0.65, which is considered substantial agreement [21]. After duplicates were removed, 1390 remaining records were screened on title and abstract, after which 1112 records were excluded. The full texts of 278 articles were assessed for eligibility. Of these, 237 articles were excluded for the reasons summarized in Figure 1 (some articles were excluded based on multiple criteria): study population (*n* = 3), NIRS-index (*n* = 47), influence extracerebral tissue not investigated (*n* = 159), no empirical peer-reviewed research (*n* = 50), or no in vivo study methodology (*n* = 14). Eight additional duplicate studies were found that had not been discovered during duplicate removal due to slight differences in title spelling. Lastly, one study only reported the measurement setup, but provided no results.

Forty-one articles were included in the review. These comprised 116 sub-studies with different NIRS devices, source–detector configurations, NIRS indices, or experimental protocols.

### 3.2. General Characteristics

An extensive overview of all study characteristics, including device type, study protocol, and results is provided in Table S2 and Table S3. Only studies with human subjects met all inclusion criteria. A total of 85 sub-studies focused on healthy participants and 31 on a patient population. Population size varied between 4 and 160 participants (median [IQR]: 12 [10,11,12,13,14,15,16,17,18,19,20,21]). All but one sub-study investigated continuous wave NIRS. Frequency-domain NIRS was assessed in one article, but this article had to be excluded post hoc because of poor study quality (as explained under Critical Appraisal). NIRS sensors were mostly placed on the forehead (*n* = 109), but a minority (*n* = 7) investigated other locations (occipital lobe, parietal lobe, or specifically covering somatosensory or motor cortex). The SDS ranged between 0.7 and 6 cm. For studies examining Hb concentrations, the majority used an SDS of 3 cm (median, IQR: 3, [3,4,5]). For the calculation of oxygen saturation indices (all included in this review as ‘rSO_2_’: regional tissue oxygen saturation), often, two or more source–detector pairs with a different SDS were used (generally, combinations of 3, 4, and 5 cm). For 23 sub-studies, no information on SDS was available.

### 3.3. Study Methodology

None of the authors directly quantified the influence of signal attenuation by the extracerebral tissue layers on the NIRS measurements, analogous to the simulation studies. Instead, the influence of the extracerebral or intracerebral tissue layers was estimated indirectly by either (1) comparing (changes in) NIRS measurements with (changes in) the measurements of intra- or extracerebral reference techniques, or (2) quantifying the change in NIRS measurements when the intra- or extracerebral tissue perfusion was selectively altered.

In total, 95 sub-studies used a methodology in which the intra and/or extracerebral perfusion was selectively altered. Intracerebral perfusion modifications were achieved by hyper-/hypocapnia (*n* = 34) or occlusion of the internal carotid artery (ICA) (*n* = 10). Extracerebral perfusion modification protocols involved scalp cuff inflation/release (*n* = 32), external carotid artery (ECA) occlusion (*n* = 7), phenylephrine infusion (*n* = 5), and several others (*n* = 7 in total). A total of 21 sub-studies applied a methodology that we classified as non-selective intra- or extracerebral perfusion modification. These were mostly cognitive or motor task studies that have a high risk of also causing systemic hemodynamic changes, particularly changes in arterial blood pressure [4].

In 68 sub-studies, an intracerebral reference technique was used; this was mostly transcranial Doppler ultrasound (TCD) (*n* = 52) or functional magnetic resonance imaging (fMRI) (*n* = 12). In one article, multiple intracerebral reference techniques were employed [23]. With TCD, the cerebral blood flow velocity (CBFv) was determined non-invasively with ultrasound in one of the large cerebral arteries, often the middle cerebral artery (MCA). With fMRI, the relative change in OxyHb and HHb concentrations in a tissue volume can be measured because of the differences in the magnetic susceptibility of the two molecules [24].

A total of 63 sub-studies used an extracerebral reference technique. The majority employed laser Doppler flowmetry (LDF) (*n* = 49), scalp pulse oximetry or photoplethysmography (PPG) (*n* = 15), or fMRI of the scalp and skull tissues (*n* = 5). Ten sub-studies applied multiple extracerebral reference techniques. LDF measures the perfusion in the microcirculation of the upper skin layers. For scalp pulse oximetry, the arterial oxygen saturation of the scalp tissue is estimated using a technique analogous to pulse oximetry performed on the finger.

### 3.4. Critical Appraisal

**Figure 2:**
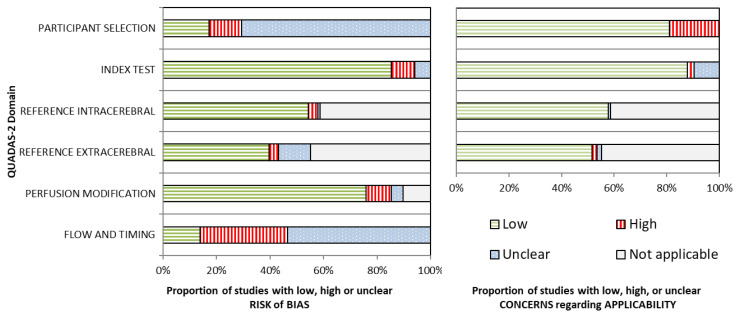
Critical appraisal results. Left panel: the proportion of 116 sub-studies that scored a low, high, or unclear risk of bias for each modified QUADAS-2 domain. Right panel: the proportion of 116 sub-studies for which there were low, high, or unclear concerns regarding applicability for each modified QUADAS-2 domain. Figure following the recommendations of Whiting and colleagues [17]. Detailed classification results are provided in Table S4. QUADAS-2, Quality Assessment of Diagnostic Accuracy Studies, revised second version.

Critical appraisal scores are summarized in Figure 2. Details are provided in Table S4.

The domains of participant selection and flow and timing showed a high or unclear risk of bias (RoB) in most articles. Specifically, participant selection was often not clearly described, and it was not always evident if a case–control design had been avoided. Exclusions were generally not clearly documented. Methods to prevent or correct artifacts were often not described. The sampling frequency of the assessed NIRS devices was mostly low or not described. No studies used a blinded study design.

If applicable, reference techniques and the perfusion modification methodology were often described well. In general, the index test (NIRS measurement) was also well described. The method of calculating Hb concentrations was mostly pre-specified. It involved application of the modified Beer–Lambert law [25], which is a standard method, but some studies used custom calculations. For rSO_2_, mostly commercially available devices were used without software changes.

Concerns regarding the applicability of the studies to the research question were low in general. Most authors included healthy participants or patients without suspected cerebrovascular diseases, which represent the general population. A minority of studies specifically included patients with cerebrovascular pathology, in whom intra- and extracerebral perfusion can deviate from that of a healthy population [26]. Some studies used manipulation of the intra- or extracerebral perfusion to calculate the investigated NIRS index, namely through clamping of the carotid artery or inflation of a cuff around the scalp. The requirement of such a protocol limits applicability to a broad population.

In total, 7 articles comprising 11 sub-studies scored a high or unclear RoB in more than 50% of QUADAS-2 domains. These articles were excluded post hoc. This cut-off for study quality was chosen because weighing individual QUADAS-2 domains is difficult, and most studies (105 sub-studies in 34 articles) would remain for data synthesis. Details are provided in Table S5 and Table S6.

### 3.5. Comparison of Haemoglobin Concentrations and Reference Techniques

**Figure 3:**
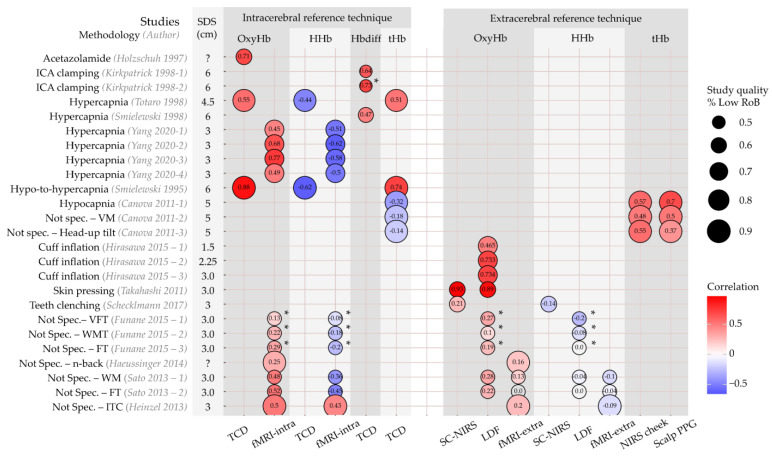
Haemoglobin concentrations; correlation between reference techniques and NIRS indices per study. Rows represent individual sub-studies. Columns have been grouped into intracerebral and extracerebral reference techniques and per NIRS index (OxyHb, HHb, Hbdiff, tHb). Circle size represents the percentage of CA domains categorized as ‘Low’ risk of bias or applicability concerns. Colour represents the correlation, with hot colours expressing positive correlation and cold colours negative correlation. Correlation is also expressed numerically in the circle. Source–detector separation (SDS) is shown in the second column. *: NIRS-index was corrected for extracerebral contamination. Extra, extracerebral; fMRI, functional magnetic resonance imaging; FT, finger tapping task, Hb, haemoglobin; Hbdiff, difference between OxyHb and HHb; HHb, deoxygenated Hb; intra, intracerebral; ITC, intertemporal choice task; LDF, laser doppler flowmetry; NIRS, near-infrared spectroscopy; Not spec., no specifically intra-or extracerebral perfusion modification; OxyHb, oxygenated Hb; PPG, photoplethysmography; RoB, risk of bias; SC, short-channel; SDS, source–detector separation; TCD, transcranial doppler; tHb, total Hb; VFT, verbal fluency task; VM, Valsalva manoeuvre; WMT, working memory task. Studies are [27,28,29,30,31,32,33,34,35,36,37,38,39,40]. Figure inspired by Bernaldo-de-Quiros and co-workers [19].

A total of 14 articles comprising 25 sub-studies compared within-subject changes in Hb concentrations (OxyHb, HHb, tHb, or Hbdiff [difference between OxyHb and HHb]) with changes in reference technique measurements [27,28,29,30,31,32,33,34,35,36,37,38,39,40]. They all expressed these relations with correlation coefficients. The correlations are illustrated in Figure 3. Study quality is reflected by circle size, itself representing the percentage of the QUADAS-2 domains classified as low RoB or applicability concerns.

Six authors used intracerebral perfusion modification protocols (acetazolamide infusion, hypercapnia, ICA clamping) and exclusively compared NIRS with intracerebral reference techniques [27,28,29,30,31,32]. Changes in OxyHb, Hbdiff, and tHb all correlated positively with changes detected by TCD and fMRI, ranging from r = 0.45 to r = 0.88. Changes in HHb were negatively correlated with changes measured with TCD and fMRI (r = −0.44 to r = −0.62). The studies using ICA clamping scored lower on study quality than studies applying hypercapnia protocols.

One author compared tHb with an intracerebral technique (TCD) and two extracerebral (NIRS on ipsilateral cheek and scalp PPG) techniques during three study protocols: hypocapnia (cerebral hypoperfusion), Valsalva manoeuvre (intracerebral hypoperfusion and extracerebral hyperperfusion), and head-up tilt (intracerebral and extracerebral hypoperfusion). During all protocols, the correlation with extracerebral techniques was higher (r = 0.37 to r = 0.7) than with intracerebral techniques (r = −0.14 to r = −0.32) [40].

Three authors applied selective extracerebral perfusion modification protocols (head-cuff inflation, teeth-clenching, skin pressure) and used only extracerebral reference techniques as comparison [33,34,35]. With cuff inflation to restrict flow to the scalp underneath the NIRS sensor, positive correlations between OxyHb and LDF-derived scalp blood flow were seen (r = 0.46 to r = 0.73) [33]. In another article, teeth-clenching was applied to change temporal muscle oxygenation with a NIRS device covering this muscle. The correlation between OxyHb and HHb with short-channel (10 mm SDS) NIRS measurements was low (OxyHb: r = 0.21; HHb: r = −0.14) [34]. Lastly, one article used the application of skin pressure in a unique protocol [35]. Changes in OxyHb and HHb were assessed in a verbal fluency protocol with and without pressure to the scalp on the measurement location. Without pressure, a clear increase in OxyHb was seen in the NIRS sensors covering the forehead. However, there were also concurrent increases in scalp perfusion as measured with both LDF and short-channel (5 mm SDS) NIRS close to the long-channel (3.0 mm SDS) NIRS optodes. A correlation of r = 0.89 between long-channel NIRS OxyHb and LDF-based scalp blood flow was found. Moreover, the correlation between long-channel OxyHb and short-channel OxyHb was r = 0.93. When the verbal fluency protocol was applied with manual pressure to the scalp, OxyHb changes were absent, as were the changes in scalp blood flow and short-channel OxyHb concentration. This may indicate that the task-related increase in OxyHb could be largely explained by changes in scalp perfusion, as measured with LDF and short-channel NIRS [35]. The authors provided no results for HHb.

Four articles comprising seven sub-studies compared changes in Hb with both intra- and extracerebral reference techniques during cognitive or motor task protocols [36,37,38,39]. These tasks were classified as no selective intracerebral or extracerebral perfusion modification. Correlations of NIRS measurements with those acquired from intra- and extracerebral reference techniques were lower than with a selective perfusion modification protocol (illustrated in Figure 3 by the paler circle colours). Absolute values ranged from |r| = 0.0 to |r| = 0.5. However, the positive correlations with OxyHb concentrations accompanied by negative correlations with HHb measurements were preserved, except for the study by Heinzel and colleagues, which found positive correlations between NIRS and fMRI measurement results for both OxyHb and HHb [39]. Study quality was generally lower for these studies.

#### 3.5.1. Extracerebral Correction Methods

Kirkpatrick and colleagues determined the correlation between TCD-based CBFv and two calculations of Hbdiff upon clamping of the internal carotid artery [32]. For one calculation, Hbdiff was corrected for changes in extracerebral perfusion by subtracting the (extrapolated) change in Hbdiff when the external carotid artery was clamped. They found a higher correlation with changes in CBFv when extracerebral correction was applied (Hbdiff: r = 0.64, Hbdiff with correction: r = 0.73).

Funane and colleagues identified ‘deep’ and ‘shallow’ OxyHb and HHb components [36]. To identify the components, groups of adjacent short (15 mm SDS) and long (30 mm SDS) NIRS channels were assessed with independent component analysis. The absolute correlation with both the intracerebral (fMRI) and extracerebral (LDF) reference technique measurements was only available for the ‘deep’ OxyHb and HHb signals, and ranged between |r| = 0.0 and |r| = 0.3.

#### 3.5.2. Source–Detector Separation (SDS)

SDS ranged between 1.5 and 6 cm (Figure 3, second column). Given the large variety of methodologies used, stratification for SDS was not performed in this review. However, changes in OxyHb for three SDS’s were compared with LDF measurements of scalp blood flow during scalp cuff inflation by Hirasawa and colleagues [33]. In contrast to the authors’ hypothesis, the correlation between OxyHb and scalp blood flow increased for a larger SDS between the NIRS optodes (1.5 cm: r = 0.47, 2.25 cm: r = 0.73, 3.0 cm: r = 0.73).

### 3.6. Comparison of Oxygen Saturation Indices and Reference Techniques

**Figure 4:**
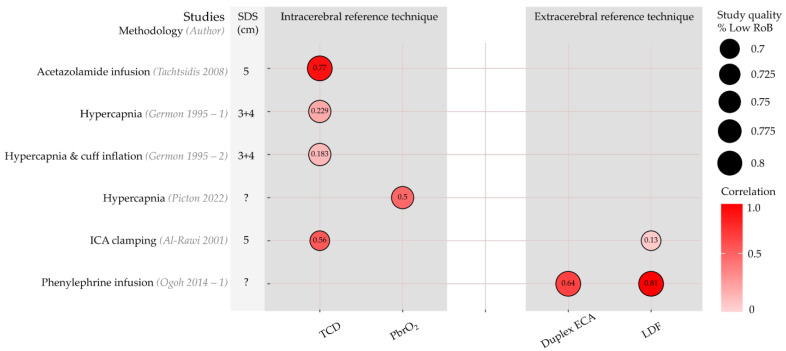
Oxygen saturation indices (rSO_2_): Correlation between reference techniques and NIRS-indices per study. Rows represent individual sub-studies. Columns have been grouped into intracerebral and extracerebral reference techniques. Circle size represents the percentage of CA domains categorized as ‘Low’ risk of bias or applicability concerns. Colour represents correlation, with hot colours expressing positive correlation and cold colours negative correlation. Correlation is also expressed numerically in the circle. Duplex ECA, duplex sonography of the external carotid artery; ICA, internal carotid artery; LDF, laser Doppler flowmetry; PbrO_2_, brain tissue oxygen; RoB, risk of bias; TCD, transcranial Doppler. Studies are [23,41,42,43,44]. Figure inspired by Bernaldo-de-Quiros and co-workers [19].

Five articles with six sub-studies compared within-subject changes in rSO_2_ with changes in reference technique measurements [23,41,42,43,44]. Correlation coefficients are shown in Figure 4. Although a high correlation was found between rSO_2_ and TCD-based CBFv upon acetazolamide infusion (r = 0.77) [41], a lower correlation with CBFv (r = 0.23) was seen during hypercapnia, which was even lower (r = 0.18) when a head-cuff was inflated to restrict perfusion to the scalp of the forehead [42]. rSO_2_ showed a good correlation with an invasive measurement of brain tissue oxygen (PbrO_2_) (r = 0.5) during hypercapnia induced by changes in ventilatory settings [43]. Upon clamping of the ICA, the correlation of changes in rSO_2_ with TCD-based CBFv was higher than for LDF-based scalp flow measurements (r = 0.56 for TCD and 0.13 for LDF) [44]. Lastly, high correlations of rSO_2_ with both duplex ECA conductance (blood flow velocity divided by mean arterial pressure) and scalp blood flow were seen during phenylephrine infusion, r = 0.64 and r = 0.81, respectively [23].

### 3.7. Studies without Within-Subject Comparisons with Reference Techniques

In 66 sub-studies in 16 articles, no reference techniques were used, or NIRS measurements were compared with those from reference techniques only on a group level and not on an individual level [40,45,46,47,48,49,50,51,52,52,53,54,55,56,57,58,59]. The results are described in detail in File S3.

## 4. Discussion

### 4.1. Main Findings

To assess the potential of NIRS as a routine intraoperative neuromonitoring modality, we here first investigated how extracerebral tissue influences NIRS measurements, and the extent of this influence, by means of a systematic review. We synthesized the results of 41 articles. NIRS measurements correlated with both intracerebral and extracerebral reference technique measurements. Although this indicates that NIRS measurements are influenced by extracerebral tissue, we could not quantify this influence.

### 4.2. Interpretation of the Results

Studies with selective intracerebral perfusion modification report high correlations (|r| = 0.23 to 0.88) between NIRS and intracerebral reference techniques. This provides evidence for the (partially) cerebral origin of the NIRS signal. However, studies using extracerebral perfusion modification showed high correlations with extracerebral reference techniques (|r| = 0.14 to 0.93). This indicates that the NIRS signal is influenced by extracerebral tissue as well. Given the high correlations with extracerebral measurements (LDF, fMRI, short-channel NIRS, Duplex ECA), it is evident that extracerebral tissue influences NIRS measurements, although a quantification of this influence was not possible based on the current literature. This applies to both Hb concentrations and rSO_2_.

Five articles compared Hb concentrations with reference techniques for both tissue layers. Four found higher correlations with intracerebral techniques during cognitive or motor tasks. One article reported higher correlations with extracerebral refence techniques using several study designs [36,37,38,39,40]. For rSO_2_, only one study performed intra- and extracerebral measurements. They found a higher correlation between NIRS measurements and intracerebral measurements during ICA clamping [44].

### 4.3. The Problem of Quantifying Extracerebral Influence

In computational simulation studies, the influence of extracerebral tissue on NIRS measurements can be quantified exactly [9]. In in vivo human studies, this has not yet been possible. Instead, the studies in this review used correlations with intra- or extracerebral reference techniques as an indirect proxy for the intra- or extracerebral influence on NIRS.

Although correlations between NIRS and reference techniques provide evidence for the influence that the intra- and extracerebral tissue have on NIRS measurements, they are insufficient to quantify this influence. If the influence of extracerebral tissue on the NIRS signal is small, changes in extracerebral perfusion would lead to relatively small changes in the NIRS measurement; however, this does not necessarily entail a low correlation. To quantify the influence of extracerebral tissue, the slope of the linear regression line through the datapoints is required, which none of the authors reported. Future research on extracerebral influence should therefore incorporate a complete regression analysis with correlation and slope.

### 4.4. Methodological Recommendations for Investigations of Extracerebral Influence

Future studies could incorporate the following methodological recommendations: (1) clearly describe participant selection and preferably study a homogenous population regarding (collateral) cerebral perfusion; (2) describe the calculation of the NIRS indices, including correction methods for extracerebral contamination; (3) apply at least one reference technique per tissue layer; (4) provide a complete description of the relation between reference techniques and NIRS measurements, including correlation coefficients, statistical significance, and regression parameters; (5) include both absolute and relative changes in NIRS indices to allow comparison between devices; (6) describe exclusions and artifact handling to prevent bias; and (7) use a standardized study protocol with both selective perfusion modification and a baseline measurement. Selective perfusion modification should only be omitted (for example, in rest) if a high signal-to-noise ratio can be expected. Otherwise, low correlations between NIRS measurements and intra- and extracerebral reference technique measurements can be expected. We advise NIRS manufacturers to validate their NIRS devices following the above recommendations to demonstrate the extent to which different tissue layers contribute to NIRS signals.

### 4.5. Implications for NIRS in Clinical Practice

NIRS devices have been used in clinical practice for many years, in particular to monitor cerebral oxygenation during cardiothoracic surgery [3,60]. Low NIRS-derived rSO_2_ values have been associated with poor clinical outcomes, and rSO_2_ optimisation management strategies may improve outcome [61,62,63,64,65,66]. The evidence for the clinical utility of NIRS measurements during cardiothoracic surgery may, however, be the result of collinearity between extracerebral and intracerebral perfusion; both skin and cerebral perfusion can be altered by systematic hemodynamic changes that may affect outcome. The question thus remains whether selective alterations in cerebral oxygen saturation will be captured with NIRS. Based on the literature discussed here, there is no indisputable evidence that most of the NIRS signal originates from intracerebral tissue. We conclude that the current uncertainty regarding the influence of extracerebral tissue on the NIRS signal remains a hurdle in the clinical implementation of NIRS as a routine intraoperative neuromonitoring modality.

### 4.6. Limitations

Although this review was performed according to the PRISMA-DTA guidelines [14] and with standardised critical appraisal, several factors limit our ability to draw strong conclusions. First, study quality varied widely; details regarding participant selection and the domain flow and timing were unclear in most articles. The number of participants was generally small (*n* < 20 for 75% of studies), which limits the generalisability of the obtained results (correlations). This furthermore limits the possibility for correction of confounders or a subgroup analysis.

Second, studies did not follow a standardized research protocol. There was large heterogeneity in study designs and outcome measurements. We therefore could not perform a quantitative (meta)analysis of the results.

Third, each of the employed reference techniques for blood flow, Hb concentration or tissue oxygen saturation in the intracerebral or extracerebral tissue layers has advantages when serving as the ‘gold standard’ for Hb concentrations and rSO_2_ as obtained with NIRS. The suitability of a reference technique depends primarily on the similarity between the measured quantities. Short-channel NIRS measures the same quantity as NIRS measurements with a longer SDS, but it is unclear how CBFv (TCD), scalp tissue blood flow (LDF), or OxyHb and HHb concentrations obtained with fMRI relate to NIRS-based Hb concentrations or rSO_2_. Reference techniques further differ in spatial resolution. fMRI offers the best spatial resolution, especially since the location of the NIRS sensors can be mapped onto the structural MRI image [36,37,38,39]. The spatial resolution of TCD is lower, as it only provides a measurement of blood flow velocity in a large cerebral blood vessel, generally the MCA. Lastly, the NIRS devices and the reference techniques strongly differ in their temporal resolution. Tissue Hb concentrations vary over time, and for a proper comparison between techniques, each technique should be able to capture the time-dependent phenomena. Cardiovascular signals may contain frequencies up to 25 Hz, implying a required sampling frequency of at least 50 Hz [67]. fMRI has a temporal resolution < 0.5 Hz, whereas LDF and TCD can reach sampling frequencies > 50 Hz. Regrettably, the sampling frequency has not been disclosed for most commercial NIRS devices.

Fourth, not all newer NIRS devices have been investigated in studies meeting our inclusion criteria. Studies that investigate the influence of extracerebral tissue on NIRS measurements from the newer devices are warranted. Furthermore, only one study specifically reported the difference between NIRS indices with and without correction for extracerebral tissue influence [32], even though several correction methods have been developed [4]. The effect of these algorithms on the extracerebral contamination of NIRS signals thus remains unclear.

Fifth, in this review, we limited our scope to continuous wave and frequency-domain NIRS, as these techniques are most commonly used in clinical practice and research. Future studies could include time-domain NIRS-measurements, for which the influence of extracerebral tissue can be lower [68].

Sixth, our initial screening criteria allowed the inclusion of large animal studies that could have been used as a model for adult human anatomy and physiology. This could have been seen as a limitation, as the validity of animal studies as a model for adult humans remains unclear. None of the authors of the animal studies identified in the initial search provided evidence of the validity of their model (porcine or ovine) for the influence of extracerebral tissue on NIRS signals in humans. In any event, during the full-text screening stage, these animal studies were all excluded because they did not meet all inclusion criteria [69,70,71,72,73,74,75]. The findings and conclusions we report are therefore not influenced by the results of animal studies.

## 5. Conclusions

Extracerebral tissue influences cerebral NIRS measurements, but there is currently insufficient evidence to quantify this influence based on the available in vivo studies. The current uncertainty regarding the influence of extracerebral tissue remains a hurdle in the clinical implementation of NIRS for intraoperative monitoring. 

## Figures and Tables

**Table 1:** Inclusion criteria for title and abstract, and full text screening. Studies were assessed in five domains: population, outcome measure, exposure, study quality and type, and study methodology. Hb, haemoglobin; HHb, deoxygenated haemoglobin; OxyHb, oxygenated haemoglobin; rSO_2_, regional tissue saturation of oxygen; StO_2_, tissue saturation of oxygen; TOI, tissue oxygenation index; TSI, tissue saturation index.

Domain	Screening Criteria		
Population	Human adults (>18 years) or adult large animal models		
Outcome measure	Cerebral continuous wave or frequency-domain near-infrared spectroscopy: OxyHb, HHb, Total Hb (tHb), Hbdiff (difference between OxyHb and HHb), or a tissue oxygen saturation index (rSO_2_). Other manufacturer-specific oxygenation indices are allowed, e.g., TOI, TSI, StO_2_.		
Exposure	Influence of extracerebral tissue (scalp, skull, cerebrospinal fluid, or dura mater) was an explicit goal of the research, i.e., it is a primary or secondary research question, or primary or secondary research goal	AND	Reference techniques for both the intra- and extracerebral tissue that measure blood flow, Hb concentrations or tissue oxygen saturation
			OR
			A perfusion modification protocol selective for the intra- or extracerebral tissue
Study quality and type	Empirical peer-reviewed research presenting original data. No reviews, book chapters, commentaries, conference abstracts without a full text, or case reports		
Study methodology	Only in vivo studies; no computational modelling, phantom, or tissue sample studies		

## Data Availability

The data presented in this study are available in Tables S2–S6. The code used for data synthesis is available in File S2.

## Supplementary Materials

The following supporting information can be downloaded at: https://www.mdpi.com/article/10.3390/jcm12082776/s1, Table S1: Critical appraisal strategy, Table S2: Detailed study characteristics haemoglobin (Hb) studies, Table S3: Detailed study characteristics oxygen saturation indices (rSO2), Table S4: Critical appraisal classification results, Table S5: Studies excluded post hoc based on study quality, Table S6: Detailed study characteristics of studies excluded post hoc, File S1: Literature search strategy, File S2: Data Synthesis Script, File S3: Results for studies without within-subject comparisons with reference techniques, File S4: PRISMA DTA Abstract Checklist, File S5: PRISMA DTA Checklist. References [76,77,78,79,80,81,82] are cited in the Supplementary Materials.
