# Supplementary material for: The Influence of Extracerebral Tissue on Continuous Wave Near-Infrared Spectroscopy in Adults: A Systematic Review of In Vivo Studies
Source: J Clin Med. 2023 Apr 8;12(8):2776. doi: 10.3390/jcm12082776 (PMC10146120; doi:10.3390/jcm12082776)
Supplement: Supplementary file 1 [file jcm-12-02776-s001.zip › File S1.pdf]

Supplementary File S1: Literature search strategy

Scopus

TITLE-ABS-KEY ("extracereb*" OR "extra cereb*" OR "extracrani*" OR "extra crani*" OR skull OR scalp) AND

TITLE-ABS-KEY ("near infrared" OR "near infra red" OR "near IR" OR "NIR" OR "NIRS" OR "fNIRS" OR "optical topography" OR "cerebral oxygenation" OR "brain oxygenation" OR "cerebral saturation" OR "brain saturation")

Embase

('skull'/exp OR 'scalp'/exp OR (‘extracereb*’ OR ‘extra cereb*’ OR ‘extracrani*’ OR ‘extra crani*’ OR skull OR scalp):ab,ti) AND

('near infrared spectroscopy'/exp OR (‘near infrared’ OR ‘near infra red’ OR ‘near IR’ OR ‘NIR’ OR ‘NIRS’ OR ‘fNIRS’ OR ‘optical topography’ OR ‘cerebral oxygenation’ OR ‘brain oxygenation’ OR ‘cerebral saturation’ OR ‘brain saturation’):ab,ti)

Web of Science

TS=("extracereb*" OR "extra cereb*" OR "extracrani*" OR "extra crani*" OR skull OR scalp) AND

TS=("near infrared" OR "near infra red" OR "near IR" OR "NIR" OR "NIRS" OR "fNIRS" OR "optical topography" OR "cerebral oxygenation" OR "brain oxygenation" OR "cerebral saturation" OR "brain saturation")

Pubmed

("Skull"[Mesh] OR "Scalp"[Mesh] OR extracereb*[tiab] OR extra cereb*[tiab] OR extracrani*[tiab] OR extra crani*[tiab] OR skull[tiab] OR scalp[tiab]) AND

("Spectroscopy, Near-Infrared"[Mesh] OR near infrared[tiab] OR near infra red[tiab] OR near IR[tiab] OR NIR[tiab] OR NIRS[tiab] OR fNIRS[tiab] OR optical topography[tiab] OR cerebral oxygenation[tiab] OR brain oxygenation[tiab] OR cerebral saturation[tiab] OR brain saturation[tiab])
