# Supplementary material for: The Influence of Extracerebral Tissue on Continuous Wave Near-Infrared Spectroscopy in Adults: A Systematic Review of In Vivo Studies
Source: J Clin Med. 2023 Apr 8;12(8):2776. doi: 10.3390/jcm12082776 (PMC10146120; doi:10.3390/jcm12082776)
Supplement: Supplementary file 1 [file jcm-12-02776-s001.zip › File S3.pdf]

File S3: Results for studies without within-subject comparisons with reference techniques

In sixty-six sub-studies in 16 articles no reference techniques were used or NIRS-measurements were compared with reference techniques only on a group level (group mean (SD) or median (IQR) values), but not on an individual level. To obtain an overview of the results from these studies, the results were first summarised. Changes in the NIRS-measurements and (if applicable) reference technique measurements were classified as ‘increased’, ‘decreased’, or ‘no change’. Changes in NIRS-measurements or reference technique measurements were classified as ‘increased’ (↑) or ’decreased (↓)’ when the study reported a ‘significant’ change, either as 1) $p<0.05$, 2) 95% confidence interval of change does not contain zero, 3) interquartile range of change does not contain zero. When this was not the case, it was classified as ‘no change’ (=). If a study reported only raw data, the non-parametric distribution was calculated post-hoc and decision was based on this distribution.

The studies were subsequently classified as 1) ‘Supports intracerebral origin of NIRS-signals’, when NIRS-measurements changed (increased or decreased) upon intracerebral perfusion modification or when the NIRS-measurements changed in the same direction as intracerebral reference technique measurements. Similarly, studies were classified as 2) ‘Supports extracerebral origin of NIRS-signals’ when NIRS-measurements changed during an extracerebral perfusion modification or when the NIRS-measurements changed in the same direction as extracerebral reference technique measurements. If both of the previous results were found, the study was classified as 3) ‘Supports both the intra- and extracerebral origin of the NIRS-signals’. If results were unclear or no changes NIRS-measurements were found, the study was classified as 4) ‘Inconclusive results’. Classification is shown in Supplementary File S3– Table 2 and Supplementary File S3 – Table 3.

Classification results are shown in Supplementary File S3 – Table 1. Studies on Hb concentrations and oxygen saturation indices generally supported the intracerebral origin of the NIRS-measurement when an intracerebral perfusion modification protocol was applied. Similarly, when the extracerebral perfusion was changed, these studies supported the extracerebral origin of the NIRS-signal. Two studies on Hb concentrations in which both the intra- and extracerebral perfusion was selectively modified, showed support for both the intra- and extracerebral origin of the NIRS-signal. For oxygen saturation indices, two studies supported the intracerebral origin. Results were inconclusive for four articles in total.

Supplementary File S3—Table S1: Classification of articles without within-subject comparisons of NIRS-measurements with reference technique measurements.

Results are divided into haemoglobin concentrations and tissue oxygen saturation indices. Rows represent the perfusion modification paradigms that were applied in each article. Columns represent the classification. Results of 16 articles are listed. In three articles both Hb concentrations and rSO_2 were studied, so the classification for Hb concentrations and rSO_2 are provided separately. Numbers indicate the number of studies in that class.

		**Supports intracerebral origin**	**Supports extracerebral origin**	**Supports both**	**Inconclusive**
**Haemoglobin concentrations**	Intracerebral perfusion modification	1 [58]	-	-	1 [47]
	Extracerebral perfusion modification	-	2 [59,60]	-	-
	Intra- and extracerebral perfusion modification	-	-	2 [56,57]	1 [49]
**Tissue oxygen saturation indices**	Intracerebral perfusion modification	3 [45–47]	-	-	-
	Extracerebral perfusion modification	-	5 [50–54,60]	-	-
	Intra- and extracerebral perfusion modification	2 [48,49]	-	-	2 [40,55]

Supplementary File S3—Table S2: Haemoglobin concentrations. Changes in haemoglobin concentrations and intracerebral and extracerebral reference techniques. Changes in NIRS-measurements or reference technique measurements were classified as ‘increased’ (↑) or ’decreased (↓)’ or ‘no change’ (=). The classification per study is shown in the last column. 1 Intra: Supports intracerebral origin. 2 Extra: Supports extracerebral origin. 3 Both: Supports both intra- and extracerebral origin. 4 Inconclusive: Inconclusive results.
ECA, external carotid artery; extra, extracerebral; ICA, internal carotid artery; Hb, haemoglobin; HHb, Deoxygenated Hb; intra, intracerebral; LDF, Laser Doppler Flowmetry; OxyHb, Oxygenated Hb; OxyHb*, Oxygenated Hb corrected for scalp blood flow; Sjv_O, jugular venous oxygen saturation; tHb, total Hb; TCD, Transcranial Doppler.

Articles	Perfusion modification	Study protocol	Hb			Reference technique intracerebral	Reference technique extracerebral	Classification
**Tateishi 1995 [58]**	Intra	Hypo- to hyper- to hypocapnia	OxyHb ↑	HHb ↓		$SjvO_2$ ↑		1 Intra
**Hirasawa 2016-1 [59]**	Extra	Cuff inflation	OxyHb ↓<br>OxyHb* =				LDF ↓	2 Extra
**Moerman 2021-1 [60]**	Extra	Phenylephrine infusion			tHb ↓			2 Extra
**Germon 1999-1-16 [56]**	Intra and extra	Hyper- to hypocapnia<br>Cuff release	OxyHb ↓<br>OxyHb ↑	HHb ↑<br>HHb ↓	tHb ↓<br>tHb =	TCD ↓		3 Both
**Germon 1998-1-4 [57]**	Intra and extra	Hyper- to hypocapnia<br>Cuff release	OxyHb ↓<br>OxyHb ↑	HHb ↑<br>HHb ↓	tHb ↓<br>tHb =	TCD ↓		3 Both
**Grubhofer 1999-1 [47]**	Intra	Hypocapnia	OxyHb =					4 Inconclusive
**Cho 1998-3+4 [49]**	Intra and extra	ICA-unclamping<br>ECA-unclamping	OxyHb↑<br>OxyHb =	HHb =<br>HHb =	tHb =<br>tHb =			4 Inconclusive

Supplementary File S3—Table S3: Oxygen saturation indices (rSO_2). Changes in rSO_2 and intracerebral and extracerebral reference techniques. Changes in NIRS-measurements or reference technique measurements were classified as ‘increased’ (↑) or ’decreased (↓)’ or ‘no change’ (=). The classification per study is shown in the last column. 1 Intra: Supports intracerebral origin. 2 Extra: Supports extracerebral origin. 4 Inconclusive: Inconclusive results. ECA, external carotid artery; extra, extracerebral; $etCO_2$, end-tidal carbon dioxide; ICA, internal carotid artery; inf, infusion; intra, intracerebral; INVOS, INVOS 4100 (Somanetics); LDF, Laser Doppler Flowmetry; NIRO, NIRO-200NX (Hamamatsu); rSO_2, regional tissue saturation of oxygen; $S_{cap}O_2$, cerebral capillary oxygen saturation; $SjvO_2$, jugular venous oxygen saturation; TCD, Transcranial Doppler.

Article	Perfusion modification	Study protocol	rSO_2	Reference technique intracerebral	Reference technique extracerebral	Classification
**Yoshitani 2002-1 [45]**	Intra	Hypocapnia	↓	TCD ↓		1 Intra
**Henson 1998 [46]**	Intra	Hypercapnia	↑	$SjvO_2$ ↑		1 Intra
**Grubhofer 1999-2 [47]**	Intra	Hypocapnia	↓			1 Intra
**Samra 1999-1+2 [48]**	Intra and extra	ICA and ECA clamping	ICA-clamping↓<br>ECA-clamping =			1 Intra
**Cho 1998-1+2 [49]**	Intra and extra	ICA and ECA unclamping	ICA-unclamping ↑<br>ECA-unclamping =			1 Intra
**Kato 2017-1/2/3/4 [50]**	Extra	Cuff inflation	↓		Scalp pulse oximetry ↓	2 Extra
**Germon 1994-1 [51]**	Extra	Cuff inflation and Frontalis muscle contraction	↓			2 Extra
**Greenberg 2016-1/2 [52,53]**	Extra	Cuff inflation	↓			2 Extra
**Davie 2012-1/2/3 [54]**	Extra	Cuff inflation	↓			2 Extra
**Moerman 2021-2 [60]**	Extra	Phenylephrine infusion	↓			2 Extra
**Canova 2011-4-6 [40]**	Intra and extra	Multiple	Protocol:<br><br>Hypocapnia ↓<br>Valsalva maneuvre ↓<br>Head-up tilt ↓	TCD<br><br>↓<br>↓<br>↓	PPG / NIRS cheek / LDF<br><br>= / = / =<br>= / ↑ / =<br>= / = / ↓	4 Inconclusive
**Sørensen 2015-1-14 [55]**	Intra and extra	Multiple	Protocol: INVOS / NIRO<br>Hypocapnia = / =<br>Phenylephrine infusion ↓ / ↓<br>Whole body heating ↑ / =<br>Noradrenaline infusion ↓ / =<br>Noradrenaline infusion + $etCO_2$ stabilisation ↓ / =<br>40° head-up tilt = / =<br>40° head-up tilt + phenylephrine infusion ↓ / ↓	TCD / $S_{cap}O_2$<br>↓ / ↓<br>= / =<br>= / =<br>= / =<br>= / =<br>= / =<br>= / =	LDF<br>=<br>↓<br>↑<br>=<br>=<br>=<br>=	4 Inconclusive
