# Supplementary material for: The Influence of Extracerebral Tissue on Continuous Wave Near-Infrared Spectroscopy in Adults: A Systematic Review of In Vivo Studies
Source: J Clin Med. 2023 Apr 8;12(8):2776. doi: 10.3390/jcm12082776 (PMC10146120; doi:10.3390/jcm12082776)
Supplement: Supplementary file 1 [file jcm-12-02776-s001.zip › Table S1.pdf]

Table S1: Critical appraisal strategy

The Quality Assessment of Diagnostic Accuracy studies, second revised version (QUADAS-2) template was tailored to fit the scope of our review, as is recommended [17], final critical appraisal strategy is shown in Table S1. The following modifications were made:

1. Split the domain Reference standard into 'Reference standard intracerebral' and 'Reference standard extracerebral'. This allowed us to individually assess each of the reference techniques used in the study.
2. Add the domain 'Perfusion modification protocol'. This allows the specific evaluating of risk of bias for the selective perfusion modification protocol, separate from other methodological concerns.
3. In the original QUADAS-2 tool, no signalling questions are present regarding applicability concerns (domains Participant selection, Index Test, Reference standard). To standardise the assessment on applicability concerns, signalling questions were added, analogous to the signalling questions for risk of bias.
4. Some signalling questions were changed or added to fit the scope of the review. These are indicated with an asterisk (*).

Table S1: Critical appraisal strategy. Modified Quality Assessment of Diagnostic Accuracy Studies 2 (QUADAS-2) criteria.

DOMAIN	*PARTICIPANT SELECTION*	*INDEX TEST*	*REFERENCE STANDARD INTRACEREBRAL*	*REFERENCE STANDARD EXTRACEREBRAL*	*PERFUSION MODIFICATION PROTOCOL*	*FLOW AND TIMING*
Risk of bias - Signalling questions: Yes/no/unclear	Was a consecutive or random sample of patients enrolled?	Were the NIRS parameters pre-specified?*	Were the intracerebral reference standard results interpreted without knowledge of the results of the index test?*	Were the extracerebral reference standard results interpreted without knowledge of the results of the index test?*	Was the protocol selective for intra- or extracerebral perfusion?*	Was there an appropriate interval between index test(s) and reference standard?
	Was a case-control design avoided?	If thresholds for alterations in NIRS-parameters were used, were these threshold pre-specified?*	Were the used intracerebral reference technique units pre-specified?*	Were the used extracerebral reference technique units pre-specified?*	Was the protocol applied consistently for all participants?*	Were sufficient measures taken to prevent (non-)physiological artefacts?*
	Did the study avoid inappropriate exclusions?	Were the index test results interpreted without knowledge of the results of the reference standard?				Did all patients receive a reference standard?
						Were all patients included in the analysis?
Risk of bias: High/low/unclear	**Could the selection of patients have introduced bias?**	**Could the conduct or interpretation of the index test have introduced bias?**	**Could the intracerebral reference standard, its conduct, or its interpretation have introduced bias?**	**Could the extracerebral reference standard, its conduct, or its interpretation have introduced bias?**	**Could the stimulation protocol have introduced bias?**	**Could the patient flow have introduced bias?**
Applicability concerns - Signalling questions: Yes/no/unclear	Can the results of the study population be generalised to a broader population?	Does the selection of NIRS-parameter influence its usability in practical use?	Does the intracerebral reference standard correctly reflect the influence of intra and/or extracerebral tissue? *	Does the extracerebral reference standard correctly reflect the influence of intra and/or extracerebral tissue?*	*Not applicable*	*Not applicable*
		Was post-processing of the NIRS-parameter applied that influences its sensitivity for extracerebral contamination?				
Applicability concerns: High/low/unclear	**Are there concerns that the included patients do not match the review question?**	**Are there concerns that the index test, its conduct, or interpretation differ from the review question?**	**Are there concerns that the target condition as defined by the reference standard does not match the review question?**	**Are there concerns that the target condition as defined by the reference standard does not match the review question?**		
