# Supplementary material for: The Influence of Extracerebral Tissue on Continuous Wave Near-Infrared Spectroscopy in Adults: A Systematic Review of In Vivo Studies
Source: J Clin Med. 2023 Apr 8;12(8):2776. doi: 10.3390/jcm12082776 (PMC10146120; doi:10.3390/jcm12082776)
Supplement: Supplementary file 1 [file jcm-12-02776-s001.zip › Table S2.pdf]

Table S2: Detailed study characteristics haemoglobin (Hb) studies

Study characteristics are provided in Table S2 for studies investigating haemoglobin (Hb) concentrations (OxyHb, HHb, tHb, Hbdiff). Studies are subdivided into sub-studies based on the following study characteristics: 1) different NIRS-devices, 2) different source-detector separations, 3) different NIRS-indices (Hb concentrations or rSO_2), 4) different experimental designs. Sub-studies are indicated with a numeral behind the author name + year (for example Hirasawa 2016-1).

Table S2: Study characteristics of studies investigating haemoglobin concentrations.
Sorted by the applied perfusion modification protocol. AU, arbitrary units; bilat, bilateral; BOLD, blood-oxygen-level-dependent (signal); CBF(v), cerebral blood flow (velocity); (et)CO_2, (end-tidal) carbon dioxide; cor, coronal; DPF, differential pathlength factor; E, extracerebral; ECA, external carotid artery; fMRI, functional magnetic resonance imaging; GM, grey matter; H, healthy participants; Hb, haemoglobin; HHb; deoxygenated Hb; I, intracerebral; ICA, internal carotid artery; IQR, inter-quartile range; L, left; lat, lateral; LDF, laser doppler flowmetry; m, muscle; MCA, middle cerebral artery; N/A, not applicable; NIRS, near-infrared spectroscopy; NS, not statistically significant; OxyHb, oxygenated Hb; P, patients; PM, perfusion modification; PPG, photoplethysmography; R, right; ref, reference technique; SD, standard deviation; SDS, source-detector separation; SE, supraorbital edge/ridge; SEM, standard error of the mean; $SjvO_2$, jugular venous oxygen saturation; SkBF, skin blood flow; sup, superior; unilat, unilateral; TCD, transcranial doppler; temp, temporal/temporalis; tHb, total Hb.
NIRS manufacturers: ETG-4000, ETG-7100, Hitachi Medical Corp; INVOS 3100, Somanetics; NIRO-500, NIRO 500, NIRO-300, NIR500, NIR1000, Hamamatsu Photonics; NIRScout XP, NIRx; FOIRE-3000, Shimadzu.

Author, Year, Sub-study	NIRS-device	SDS (cm)	Sensor location	Pop	n	Ref I	Ref E	PM	PM method	Methodology details	Results
**Holzschuh 1997 [29]**	INVOS 3100	?	Forehead	P	21	Xenon133 CBF	N/A	I	Acetazolamide infusion	1000 mg bolus 15 min before measurement	Correlation(ΔCBF, ΔNIRS): r= 0.71, p<0.05.<br>n=18: CBF (mean (SEM)): 41.1 (2.1) to 53.7 (3.1) ml/100g/min (30.8%).<br>OxyHb (mean (SEM)): 69.2 (1.6) to 72.5 (1.4)% (4.7%)<br>n=3: CBF: 'decrease of 12.6%', OxyHb: In n=2 a decrease of 1.9%, n=1 increase of 4.9%)
**Germon 1999-1 [56]**	Prototype	0.7	L side forehead above temp. crest, avoiding m. temp.	H	10	TCD	N/A	I	Hyper- to hypocapnia	5% CO_2 inhalation to stable etCO_2, then hyperventilation to metronome to stable etCO_2	CBFv in MCA: 75.3 (10) to 39.6 (9) cm/s (p<0.001)<br>NIRS (mean, IQR) (estimated from graphs): SDS 0.7 cm: OxyHb: -0.00 (-0.01, 0.00), HHb: 0.00 (0.00,0.01), tHb: 0.00 (-0.01,0.01) μM *total path length μM
**Germon 1999-2 [56]**	Prototype	1.3	L side forehead above temp. crest, avoiding m. temp.	H	10	TCD	N/A	I	Hyper- to hypocapnia	5% CO_2 inhalation to stable etCO_2, then hyperventilation to metronome to stable etCO_2	NIRS (mean, IQR) (estimated from graphs): SDS 1.3 cm: OxyHb: -0.01 (-0.03, 0.01), HHb: 0.01 (0.01,0.02), tHb: 0.01 (-0.01,0.01) μM *total path length
**Germon 1999-3 [56]**	Prototype	2	L side forehead above temp. crest, avoiding m. temp.	H	10	TCD	N/A	I	Hyper- to hypocapnia	5% CO_2 inhalation to stable etCO_2, then hyperventilation to metronome to stable etCO_2	NIRS (mean, IQR) (estimated from graphs): SDS 2.0 cm: OxyHb: -0.02 (-0.04, 0.01), HHb: 0.02 (0.01,0.03), tHb: 0.00 (-0.02,0.02) μM *total path length
**Germon 1999-4 [56]**	Prototype	2.7	L side forehead above temp. crest, avoiding m. temp.	H	10	TCD	N/A	I	Hyper- to hypocapnia	5% CO_2 inhalation to stable etCO_2, then hyperventilation to metronome to stable etCO_2	NIRS (mean, IQR) (estimated from graphs): SDS 2.7 cm: OxyHb: -0.04 (-0.07, 0.02), HHb: 0.03 (0.02,0.05), tHb: -0.01(-0.02,0.03) μM *total path length
**Germon 1999-5 [56]**	Prototype	3.4	L side forehead above temp. crest, avoiding m. temp.	H	10	TCD	N/A	I	Hyper- to hypocapnia	5% CO_2 inhalation to stable etCO_2, then hyperventilation to metronome to stable etCO_2	NIRS (mean, IQR) (estimated from graphs): SDS 3.4 cm: OxyHb: -0.07(-0.09,-0.00), HHb: 0.05(0.03,0.07), tHb: -0.01(-0.03,0.03) μM *total path length
**Germon 1999-6 [56]**	Prototype	4.1	L side forehead above temp. crest, avoiding m. temp.	H	10	TCD	N/A	I	Hyper- to hypocapnia	5% CO_2 inhalation to stable etCO_2, then hyperventilation to metronome to stable etCO_2	NIRS (mean, IQR) (estimated from graphs): SDS 4.1 cm: OxyHb: -0.09(-0.12,-0.05), HHb: 0.07(0.05,0.10), tHb: -0.02(-0.05,0.02) μM *total path length
**Germon 1999-7 [56]**	Prototype	4.8	L side forehead above temp. crest, avoiding m. temp.	H	10	TCD	N/A	I	Hyper- to hypocapnia	5% CO_2 inhalation to stable etCO_2, then hyperventilation to metronome to stable etCO_2	NIRS (mean, IQR) (estimated from graphs): SDS 4.8 cm: OxyHb: -0.14(-0.17,-0.12), HHb: 0.09(0.07,0.11), tHb: -0.05(-0.07,-0.03) μM *total path length
**Germon 1999-8 [56]**	Prototype	5.5	L side forehead above temp. crest, avoiding m. temp.	H	10	TCD	N/A	I	Hyper- to hypocapnia	5% CO_2 inhalation to stable etCO_2, then hyperventilation to metronome to stable etCO_2	NIRS (mean, IQR) (estimated from graphs): SDS 5.5 cm: OxyHb: -0.19(-0.22,-0.15), HHb: 0.11(0.07,0.15), tHb: -0.07(-0.12,-0.04) μM *total path length
**Germon 1998-1** [57]	Prototype	5.5	Transmitter med. to L sup. temp. line, as close to cor. suture as hairline allowed. Detectors antero-medially	H	10	TCD	N/A	I	Hyper- to hypocapnia	5% CO_2 inhalation to stable etCO_2, then hyperventilation to metronome to stable etCO_2	CBFv: '44% decrease MCA velocity', 69.1 (SD 9) to 38.7 (SD 7) cm/s p<0.001<br>ΔOxyHb (5.5 cm) (mean, IQR) : -0.08 (-0.12, -0.6) μM *total path length (estimated from graphs)<br>ΔHHb (5.5 cm): 0.06 (0.055, 0.07) μM *total path length (estimated from graphs)
**Germon 1998-2 [57]**	Prototype	2.7	Transmitter med. to L sup. temp. line, as close to cor. suture as hairline allowed. Detectors antero-medially	H	10	TCD	N/A	I	Hyper- to hypocapnia	5% CO_2 inhalation to stable etCO_2, then hyperventilation to metronome to stable etCO_2	CBFv: '44% decrease MCA velocity', 69.1 (SD 9) to 38.7 (SD 7) cm/s p<0.001<br>ΔOxyHb (2.7 cm): -0.07 (-0.10, -0.04)μM *total path length (estimated from graphs)<br>ΔHHb (2.7 cm): 0.045 (0.04, 0.06) μM *total path length (estimated from graphs)
**Totaro 1998 [27]**	NIRO-500	4.5	R frontal region. Receiver 2 cm from midline, 2 cm above SE. Transmitter at 4.5 cm along hairline	H	34	TCD	N/A	I	Hypercapnia	5% CO_2 inhalation for 3 min	Correlation (ΔOxyHb, ΔCBFv): r=0.55 (p<0.001)<br>Correlation (ΔHHb, ΔCBFv): r=-0.44 (p<0.01)<br>Correlation(ΔtHb, ΔCBFv): r=0.51 (p<0.001)
**Smielewski 1998 [30]**	NIRO-500	6	Forehead, ipsilateral to TCD	P	160	TCD	N/A	I	Hypercapnia	5% CO_2 inhalation for 5 min	NIRS reactivity (ΔNIRS per ΔetCO_2 of 1kPa) vs TCD reactivity (ΔCBFv per ΔetCO_2 of 1kPa)<br>OxyHb: Not described, HHb: Not described, Hbdiff: 'Highest, correlation r=0.47, p<0.000001'<br>Temporal artery compression: No results ΔLDF versus ΔNIRS, but 'skin contribution to HbO_2 reactivity is -11 to 105%, median 16.5%'
**Yang 2020-1 [28]**	NIRScoutXP	3	Over prefrontal region of brain	H	10	fMRI	N/A	I	Hypercapnia	2 2-min blocks of CO_2 inhalation. CO_2 concentrations computer controlled to etCO_2 10 mmHg above baseline	Correlation(ΔBOLD, ΔOxyHb): r=0.45 (SD 0.44), p<0.001 averaged over 17 channels)<br>Correlation(ΔBOLD, ΔHHb): r=-0.51 (SD 0.32), p<0.001 averaged over 17 channels)
**Yang 2020-2 [28]**	NIRScoutXP	3	Over prefrontal region of brain	H	4	fMRI	N/A	I	Hypercapnia	2 3-min blocks of CO_2 inhalation. CO_2 concentrations computer controlled. etCO_2 slowly ramped to 10 mmHg above baseline	Correlation(ΔBOLD, ΔOxyHb): r=0.68 (SD 0.06), p<0.001 averaged over 17 channels)<br>Correlation(ΔBOLD, ΔHHb): r=-0.62 (SD 0.15), p<0.005 averaged over 17 channels)
**Yang 2020-3 [28]**	NIRScoutXP	3	Over prefrontal region of brain	H	4	fMRI	N/A	I	Hypercapnia	6 epochs of 18-s paced breathing to 20-s breath holding	Correlation(ΔBOLD, ΔOxyHb): r=0.77 (SD 0.1), p<0.005 averaged over 17 channels)<br>Correlation(ΔBOLD, ΔHHb): r=-0.58 (SD 0.11), p<0.005 averaged over 17 channels)
**Yang 2020-4 [28]**	NIRScoutXP	3	Over prefrontal region of brain	H	4	fMRI	N/A	I	Hypercapnia	6 epochs of 18-s rest to 20s CO_2 inhalation.CO_2 concentrations computer controlled to etCO_2 10 mmHg above baseline	Correlation(ΔBOLD, ΔOxyHb): r=0.49 (SD 0.29), p<0.05 averaged over 17 channels)<br>Correlation(ΔBOLD, ΔHHb): r=-0.50 (SD 0.34), p<0.05 averaged over 17 channels)

**Smielewski 1995 [31]**	NIR 1000	6	Frontal region, receiver 2 cm lat. to midline, 2 cm above SE. Transmitter toward hairline	H	50	TCD	LDF	I	Hypo- to hyper- to hypocapnia	5 min hyperventilation, 5 min rest, 5 min increased CO_2 inhalation, 3 min rest, 3 min hyperventilation	Correlation(ΔOxyHb, ΔCBFv): r=0.88 (p<0.001)<br>Correlation(ΔHHb ΔCBFv): r=-0.62 (p<0.001)<br>Correlation(ΔtHb, ΔCBFv): r=0.74 (p<0.001)<br>Correlation(Δ'NIRS', ΔLDF) = p>0.35
**Tateishi 1995 [58]**	NIRO-500	4	Upper forehead, 2 and 6 cm from midline	P	9	$SjvO_2$	N/A	I	Hypo- to hyper- to hypocapnia	Ventilated P: hyperventilation for 30 min to 1 hour, then increase of $PaCO_2$ (CO_2 inhalation and Ventilatory rate reduction), then return to hyperventilation	$SjvO_2$ (mean+-SEM): 63+-3% to 76+-3%<br>OxyHb (mean+-SEM): 3.5+-0.9 μM (significant change)<br>HHb (mean+-SEM): -1.5+-0.4 μM<br>No quantitative comparison reported: For n=9/10: 'similar slope of ΔOxyHb versus slope $\Delta SjvO_2$'. For n=1/10: 'negligible ΔOxyHb while increase $SjvO_2$ of 20%.
**Grubhofer 1999-1 [47]**	NIRO-500	6	R or L forehead	H	15	N/A	N/A	I	Hypocapnia	Hyperventilation twice rest respiratory rate to $etCO_2$ < 20 mmHg	OxyHb: 'insignificant changes'. Correlation $etCO_2$-OxyHb: r= 0.05, p=0.49<br>HHb: +1.61+-0.48 μM. Correlation $etCO_2$-HHb : r=0.01, p=0.85
**Canova 2011-1 [40]**	NIRO-300	5	High on L forehead excluding m. temp., lat. to midline to exclude sup sag sinus	H	22	TCD	PPG, NIRS on cheek, LDF	I	Hypocapnia	Hyperventilation with visual feedback to $etCO_2$ = 20 mmHg	Correlation(ΔtHb, ΔCBFv): r=-0.32 (NS), ΔCBFv: -23.63+-14.46 % (p<0.01)<br>Correlation(ΔtHb, ΔPPG): r=0.70 (p<0.01), ΔPPG: 0.71+-2.07 AU (NS),<br>Correlation(ΔtHb, ΔtHb on cheek): 0.57 (p<0.01) , ΔtHb on cheek: 1.17+-3.73 muM (NS)<br>ΔLDF: 32.89+-43.30 % (NS), n=9, no correlations available<br>ΔtHb: 0.48+-1.97 muM (NS)<br>ΔABP: -4.10 +-7.53%
**Canova 2011-2 [40]**	NIRO-300	5	High on L forehead excluding m. temp., lat. to midline to exclude sup sag sinus	H	24	TCD	PPG, NIRS on cheek, LDF	No	No	Valsalva maneuvre: Holding positive alveolar pressure of 40mmHg for 15s. Visual feedback + small leak prevented closing glottis	Correlation(ΔtHb, ΔCBFv): r=-0.18 (NS), ΔCBFv: -6.75+-7.78 % (p<0.01)<br>Correlation(ΔtHb, ΔPPG): r=0.50 (p<0.01), ΔPPG: 3.74+-3.02 AU (p<0.01),<br>Correlation(ΔtHb, ΔtHb on cheek): 0.48 (p<0.05) , ΔtHb on cheek: 16.13+-8.81 muM (p<0.01)<br>ΔLDF: -16.71 +-37.00 % (NS), n=9, no correlations available<br>ΔtHb: 6.73 +- 3.49 muM (p<0.01)<br>ΔABP: 4.84 +-11.44 % (NS)
**Canova 2011-3 [40]**	NIRO-300	5	High on L forehead excluding m. temp., lat. to midline to exclude sup sag sinus	H	22	TCD	PPG, NIRS on cheek, LDF	No	No	Head-up tilt to 70 degrees for 5 min	Correlation(ΔtHb, ΔCBFv): r=-0.14 (NS), ΔCBFv: -6.23+-6.10 % (p<0.05)<br>Correlation(ΔtHb, ΔPPG): r=0.55 (p<0.01), ΔPPG: -0.58+-3.30 AU (NS),<br>Correlation(ΔtHb, ΔtHb on cheek): 0.37 (NS), ΔtHb on cheek: -4.75 +-3.52 muM (NS)<br>ΔLDF: -27.47 +-13.24 % (p<0.01), n=9, no correlations available<br>ΔtHb: 0.77+-3.68 muM (NS)<br>ΔABP: 3.71+-19.52 % (NS)
**Kirkpatrick 1998-1** [32]	NIR500	6	Unilat., receiver 2 cm lat. to midline and 2 cm cor. to SE. Transmitter toward cor. suture	P	76	TCD	LDF	I	ICA clamping	ICA clamp 2 min after ECA clamp	Correlation (ΔTotalHbdiff, ΔCBFv): r = 0.64, p 0.001.<br>No ΔTotalHbdiff-derived threshold for Severe Cerebral Ischaemia present
**Kirkpatrick 1998-2** [32]	NIR500	6	Unilat., receiver 2 cm lat. to midline and 2 cm cor. to SE. Transmitter toward cor. suture	P	76	TCD	LDF	I	ICA clamping	ICA clamp 2 min after ECA clamp	NIRS corrected for extracranial changes: Correlation (ΔICAHbdiff, ΔCBFv): r= 0.73, p 0.0001.<br>ΔICAHbdiff <5 μM: 100% specificity and 93% specificity for absence of Severe Cerebral Ischaemia.<br>ΔICAHbdiff >6.8 μM: 100% specificity and 81% sensitivity for presence of Severe Cerebral Ischaemia.
**Cho 1998-2** [49]	NIRO-500	?	As much as possible over MCA territory, as close as possible to frontal hairline, avoiding midline	P	20	N/A	N/A	I	ICA unclamping	ICA unclamping 30-60 sec after ECA unclamping	ICA unclamping: ΔHbO_2 = 13.76 (SD 12.76) μM<br>'No changes HHb and THb during ICA unclamping'
**Hirasawa 2016-1 [59]**	ETG-7100	3.0	L on forehead above SE	H	12	N/A	LDF	E	Cuff inflation	Inflation to 80 mmHg for 5 sec	OxyHb (mean+-SD): -0.43 +-0.2 mM*mm (estimated from graph)<br>LDF (AU, (mean+-SD)): -100 +- 50 AU (estimated from graph)
**Hirasawa 2016-2 [59]**	ETG-7100	3.0	L on forehead above SE	H	12	N/A	LDF	E	Cuff inflation	Inflation to 80 mmHg for 5 sec	OxyHb with scalp correction (mean+-SD): No significant change<br>LDF (AU, (mean+-SD)): -100 +- 50 AU (estimated from graph)
**Hirasawa 2015-1 [33]**	ETG-7100	1.5	L on forehead above SE	H	7	N/A	LDF	E	Cuff inflation	30 sec cuff inflation-deflation to 60 mmHg	OxyHb (SDS 15mm) (mean+-SD): -0.45+-0.2 mM*mm<br>HHb (mean+-SD): -0.036+-0.042 mM*mm<br>SkBF (AU) (mean+-SD): mean+-SD: -131+-66 AU
**Hirasawa 2015-2** [33]	ETG-7100	1.5	L on forehead above SE	H	7	N/A	LDF	E	Cuff inflation	30 sec cuff inflation-deflation to 80 mmHg	OxyHb (SDS 15mm) (mean+-SD): -0.7+-0.3 mM*mm<br>HHb (mean+-SD): -0.090 +-0.086 mM*mm<br>SkBF (AU) (mean+-SD): mean+-SD: -212+-125 AU
**Hirasawa 2015-3** [33]	ETG-7100	2.25	L on forehead above SE	H	7	N/A	LDF	E	Cuff inflation	30 sec cuff inflation-deflation to 60 mmHg	OxyHb (SDS 22.5 mm) (mean+-SD): -0.6+-0.3 mM*mm<br>HHb (mean+-SD): -0.00+-0.10 mM*mm<br>SkBF (AU) (mean+-SD): mean+-SD: -131+-66 AU
**Hirasawa 2015-4** [33]	ETG-7100	2.25	L on forehead above SE	H	7	N/A	LDF	E	Cuff inflation	30 sec cuff inflation-deflation to 80 mmHg	OxyHb (SDS 22.5 mm) (mean+-SD): -0.8+-0.3 mM*mm<br>HHb (mean+-SD): -0.036+-0.17 mM*mm<br>SkBF (AU) (mean+-SD): mean+-SD: -212+-125 AU

**Hirasawa 2015-5 [33]**	ETG-7100	3.0	L on forehead above SE	H	7	N/A	LDF	E	Cuff inflation	30 sec cuff inflation-deflation to 60 mmHg	OxyHb (SDS 30 mm) (mean+-SD): -0.55+-0.3 mM*mm<br>HHb (mean+-SD): -0.042 +-0.11 mM*mm<br>SkBF (AU) (mean+-SD): mean+-SD: -131+-66 AU
**Hirasawa 2015-6 [33]**	ETG-7100	3.0	L on forehead above SE	H	7	N/A	LDF	E	Cuff inflation	30 sec cuff inflation-deflation to 80 mmHg	OxyHb (SDS 30 mm) (mean+-SD): -0.8+-0.35 mmHg (estimated from graph)<br>HHb (mean+-SD): -0.00+-0.11 mM*mm<br>SkBF (AU) (mean+-SD): mean+-SD: -212+-125 AU
**Hirasawa 2015-7 [33]**	ETG-7100	1.5	L on forehead above SE	H	7	N/A	LDF	E	Cuff inflation	Average of 30 sec cuff inflation-deflation to 20, 40, 60, 80 mmHg	Correlation OxyHb-LDF for all cuff pressures: OxyHb SDS 15 mm - LDF: r=0.465, p=0.013
**Hirasawa 2015-8 [33]**	ETG-7100	2.25	L on forehead above SE	H	7	N/A	LDF	E	Cuff inflation	Average of 30 sec cuff inflation-deflation to 20, 40, 60, 80 mmHg	Correlation OxyHb-LDF for all cuff pressures: OxyHb SDS 22.5 mm - LDF: r=0.733, p<0.001
**Hirasawa 2015-9 [33]**	ETG-7100	3.0	L on forehead above SE	H	7	N/A	LDF	E	Cuff inflation	Average of 30 sec cuff inflation-deflation to 20, 40, 60, 80 mmHg	Correlation OxyHb-LDF for all cuff pressures: OxyHb SDS 30 mm - LDF: r=0.734, p<0.001
**Germon 1999-9 [56]**	Prototype	0.7	L side forehead above temp. crest, avoiding m. temp.	H	10	N/A	N/A	E	Cuff release	Inflation to 200 mmHg for 3 min, then release for 3 min	No significant changes in systemic phsyiological variables'<br>NIRS (mean, IQR) (estimated from graphs): SDS 0.7 cm: OxyHb: 0.06 (0.02,0.09), HHb: -0.01 (-0.02,-0.00), tHb: 0.05 (0.00,0.07)
**Germon 1999-10 [56]**	Prototype	1.3	L side forehead above temp. crest, avoiding m. temp.	H	10	N/A	N/A	E	Cuff release	Inflation to 200 mmHg for 3 min, then release for 3 min	NIRS (mean, IQR) (estimated from graphs): SDS 1.3 cm: OxyHb: 0.10 (0.04,0.22), HHb: -0.03 (-0.03,-0.01), tHb: 0.09 (-0.00,0.17)
**Germon 1999-11 [56]**	Prototype	2.0	L side forehead above temp. crest, avoiding m. temp.	H	10	N/A	N/A	E	Cuff release	Inflation to 200 mmHg for 3 min, then release for 3 min	NIRS (mean, IQR) (estimated from graphs): SDS 2.0 cm: OxyHb: 0.14 (0.06,0.26), HHb: -0.03 (-0.05,-0.01), tHb: 0.08 (-0.00,0.19)
**Germon 1999-12 [56]**	Prototype	2.7	L side forehead above temp. crest, avoiding m. temp.	H	10	N/A	N/A	E	Cuff release	Inflation to 200 mmHg for 3 min, then release for 3 min	NIRS (mean, IQR) (estimated from graphs): SDS 2.7 cm: OxyHb: 0.15 (-0.01,0.28), HHb: -0.05 (-0.06,-0.01), tHb: 0.10 (-0.06,0.18)
**Germon 1999-13 [56]**	Prototype	3.4	L side forehead above temp. crest, avoiding m. temp.	H	10	N/A	N/A	E	Cuff release	Inflation to 200 mmHg for 3 min, then release for 3 min	NIRS (mean, IQR) (estimated from graphs): SDS 3.4 cm: OxyHb: 0.14(0.04,0.29), HHb: -0.05(-0.07,-0.01), tHb: 0.10 (-0.03,0.17)
**Germon 1999-14 [56]**	Prototype	4.1	L side forehead above temp. crest, avoiding m. temp.	H	10	N/A	N/A	E	Cuff release	Inflation to 200 mmHg for 3 min, then release for 3 min	NIRS (mean, IQR) (estimated from graphs): SDS 4.1 cm: OxyHb: 0.14(0.04,0.31), HHb: -0.05(-0.07,-0.02), tHb: 0.08 (-0.07,0.18)
**Germon 1999-15 [56]**	Prototype	4.8	L side forehead above temp. crest, avoiding m. temp.	H	10	N/A	N/A	E	Cuff release	Inflation to 200 mmHg for 3 min, then release for 3 min	NIRS (mean, IQR) (estimated from graphs): SDS 4.8 cm: OxyHb: 0.16(0.07,0.36), HHb: -0.04(-0.07,-0.03), tHb: 0.07 (0.04,0.24)
**Germon 1999-16 [56]**	Prototype	5.5	L side forehead above temp. crest, avoiding m. temp.	H	10	N/A	N/A	E	Cuff release	Inflation to 200 mmHg for 3 min, then release for 3 min	NIRS (mean, IQR) (estimated from graphs): SDS 5.5 cm: OxyHb: 0.13(0.07,0.24), HHb: -0.05(-0.10,-0.03), tHb: 0.12 (0.00,0.21)
**Germon 1998-3 [57]**	Prototype	5.5	Transmitter medial to L sup. temp. line, as close to cor. suture as hairline allowed. Detectors antero-medially	H	10	N/A	N/A	E	Cuff release	Inflation to 200 mmHg for 7 min, then release for 3 min	ΔOxyHb (5.5 cm)(median +-IQR): 60 (40, 65) µM*DPF (estimated from graphs)<br>ΔHHb (5.5 cm (median +-IQR)): 120 (80, 130) µM*DPF (estimated from graphs)
**Germon 1998-4 [57]**	Prototype	2.7	Transmitter medial to L sup. temp. line, as close to cor. suture as hairline allowed. Detectors antero-medially	H	10	N/A	N/A	E	Cuff release	Inflation to 200 mmHg for 7 min, then release for 3 min	ΔOxyHb (2.7 cm) (median +-IQR)): 15 (10, 30) mM*DPF (estimated from graphs)<br>ΔHHb (2.7 cm)(median +-IQR): 0 (-5, 10) µM*DPF (estimated from graphs)
**Takahashi 2011 [35]**	FOIRE-3000	3.0	Frontal pole and bilat. fronto-temp. region	H	46	N/A	NIRS-SC, LDF	E	Skin pressing	Manual pressure with custom rubber block over NIRS-sensor during verbal fluency task	Verbal fluency task: Correlation LDF and OxyHb: r¬=0.89. Correlation SC-NIRS (5 mm) and OxyHb: r =0.93<br>Verbal fluency task during skin pressure: NIRS optodes underneath rubber block: ‘No task-related increase in OxyHb and decrease in HHb’ Concentrations OxyHb and HHb not difference from baseline (p=0.46)<br>NIRS optodes left/right from rubber block: 'Increase in OxyHb and decrease in HHb remained almost unchanged’<br>LDF Left/right from rubber block: 'Increase remained almost unchanged’
**Cho 1998-1 [49]**	NIRO-500	?	As much as possible over MCA territory, as close as possible to frontal hairline, avoiding midline	P	20	N/A	N/A	E	ECA unclamping	ECA unclamping 30-60 sec before ICA unclamping	ECA unclamping: ΔHbO_2 = 1.29 (SD 4.42) µM<br>‘No changes HHb and THb during ECA unclamping’
**Moerman 2021-1 [60]**	NIRO-200NX	?	L and R forehead	P	20	N/A	N/A	E	Phenylephrine infusion	100 µg whenever clinically indicated	tHb pre-phenylephrine: -5.10 IQR -11.81 to 0.62<br>tHb post-phenylephrine: -6.36 IQR -11.69 to 0.25<br>Difference (Hodges-Lehmann method): -0.415 95%CI − 0.665 to − 0.205 (p < 0.001)
**Schecklmann 2017 [34]**	ETG-4000	3	L side of the forehead	H	31	N/A	NIRS-SC	E	Teeth clenching	3 times 2-sec teeth clenching	Sign. changes over temple area: increase OxyHb and decrease HHb 3-34 sec after clenching.<br>Correlations SOD-LOD NIRS post-clenching:<br>OxyHb: average: r=0.21, p=0.27, peak: r=0.02, p=0.92, peak latency: r=0.43, p<0.05<br>HHb: average: r=-0.14, p=0.45, peak: r=-0.17, p=0.35, peak latency: r=-0.41, p<0.05
**Funane 2015-1 [36]**	ETG-4000	3.0	Covering L prefrontal cortex and L somatosensory or motor cortex	H	12	fMRI	LDF	No	No	Verbal fluency task for 60 sec	Correlation coefficients (Fisher's Z) NIRS versus LDF<br>OxyHb: original 0.3, deep 0.2, shallow 0.4; HHb: original 0.28, deep 0.2, shallow 0.31.<br>Correlation coefficients (Fisher's Z) NIRS versus GM-BOLD<br>OxyHb: VFT: deep 0.13 ; shallow 0.1; HHb: VFT: deep 0.08 ; shallow 0.03. Not available for ‘original’ signals

**Funane 2015-2 [36]**	ETG-4000	3.0	Covering L prefrontal cortex and L somatosensory or motor cortex	H	12	fMRI	LDF	No	No	Working memory task with 1.5 sec stimulus and 7 sec delay	Correlation coefficients (Fisher's Z) NIRS versus LDF<br>OxyHb: original 0.15, deep 0.1, shallow 0.19; HHb: original 0.15, deep 0.08, shallow 0.19.<br>Correlation coefficients (Fisher's Z) NIRS versus GM-BOLD<br>OxyHb: deep 0.22 ; shallow 0.2; HHb: deep 0.19 ; shallow 0.1. Not available for 'original' signals
**Funane 2015-3 [36]**	ETG-4000	3.0	Covering L prefrontal cortex and L somatosensory or motor cortex	H	12	fMRI	LDF	No	No	Thumb to finger tapping, alternating between fingers for 15 sec	Correlation coefficients (Fisher's Z) NIRS versus LDF<br>OxyHb: original 0.27, deep 0.2, shallow 0.3; HHb: original 0.02, deep 0.01, shallow 0.02.<br>Correlation coefficients (Fisher's Z) NIRS versus GM-BOLD<br>OxyHb: deep 0.3 ; shallow 0.25; HHb deep 0.22 ; shallow 0.18. Not available for 'original' signals
**Haeussinger 2014 [37]**	ETG-4000	?	R frontal side of head	H	24	fMRI	fMRI	No	No	1-back and 2 back task	Correlations between raw OxyHb and skin-BOLD: r=0.16<br>Correlations between raw OxyHb and GM-BOLD: r=0.25
**Sato 2013-1 [38]**	ETG-4000	3.0	Probe 1: Wide area on forehead. Probe 2: L prefrontal and parietal head	H	19	fMRI	fMRI, LDF	No	No	Working memory task with 1.5 sec stimulus and 7 sec delay	Correlation (Z-value) fMRI (Gray matter) mean+-SD<br>OxyHb: 0.52 +-0.28 (SD)<br>HHb: -0.38 +- 0.37 (SD)<br>Correlation (Z-value) fMRI (soft tissue extracerebral) mean+-SD<br>OxyHb: 0.13+-0.22 (SD) (NS)<br>HHb: -0.10+-0.20 (SD) (NS)<br>Correlation (Z-value) LDF mean+-SD<br>OxyHb: 0.29+-0.23<br>HHb: -0.04+-0.16 (NS)
**Sato 2013-2 [38]**	ETG-4000	3.0	Probe 1: Wide area on forehead. Probe 2: L prefrontal and parietal head	H	16	fMRI	fMRI, LDF	No	No	Thumb to finger tapping, alternating between fingers for 15 sec	Correlation (Z-value) fMRI (Gray matter) mean+-SD<br>OxyHb: 0.57 +-0.47 (SD)<br>HHb: -0.49 +- 0.38 (SD)<br>Correlation (Z-value) fMRI (soft tissue extracerebral) mean+-SD<br>OxyHb: 0.0+-0.27 (SD) (NS)<br>HHb: -0.04+-0.21 (SD) (NS)<br>Correlation (Z-value) LDF mean+-SD<br>OxyHb: 0.22+-0.29 (SD) (NS)<br>HHb: -0.0+-0.20 (SD) (NS)
**Heinzel 2013** [39]	ETG-4000	3	R prefrontal cortex: Bottom optodes on line from Fpz to F8.	H	20	fMRI	fMRI	No	No	Inter temporal choice paradigm, with valuation of monetary reward options	Channel-wise correlation coefficient between raw OxyHb and GM-BOLD : r= 0.50, p<0.05<br>Channel-wise correlation coefficient between raw HHb and GM-BOLD: r=0.43, p<0.05<br>Correlation coefficient between raw OxyHb and scalp-BOLD (temple region): r < 0.2<br>Correlation coefficient between raw HHb and scalp-BOLD (temple region): r > -0.09<br>Correlation coefficient between raw OxyHb and scalp-BOLD (middle frontal gyrus): r<0.1<br>Correlation coefficient between raw HHb and scalp-BOLD (temple region): r<0.12<br>OxyHb-scalp cortex distance: Up to 6% of variance explained<br>HHb-scalp cortex distance: Up to 20% of variance explained
