# Supplementary material for: The Influence of Extracerebral Tissue on Continuous Wave Near-Infrared Spectroscopy in Adults: A Systematic Review of In Vivo Studies
Source: J Clin Med. 2023 Apr 8;12(8):2776. doi: 10.3390/jcm12082776 (PMC10146120; doi:10.3390/jcm12082776)
Supplement: Supplementary file 1 [file jcm-12-02776-s001.zip › Table S3.pdf]

Table S3: Detailed study characteristics oxygen saturation indices (rSO_2)

Study characteristics are provided in Table S3 for studies investigating oxygen saturation indices (rSO_2). Studies are subdivided into sub-studies based on the following study characteristics: 1) different NIRS-devices, 2) different source-detector separations, 3) different NIRS-indices (Hb concentrations or rSO_2), 4) different experimental designs. Sub-studies are indicated with a numeral behind the author name + year (for example Hirasawa 2016-1).

Table S3: Study characteristics of studies investigating oxygen saturation indices (rSO_2).
Sorted by the applied perfusion modification protocol. bilat, bilateral; CBF(v), cerebral blood flow (velocity); CO_2, carbon dioxide; E/Extra, extracerebral; ECA, external carotid artery; H, healthy participants; I/Intra, intracerebral; ICA, internal carotid artery; IQR, inter-quartile range; L, left; lat, lateral; LDF, laser doppler flowmetry; m, muscle; MCA, middle cerebral artery; N/A, not applicable; NIRS, near-infrared spectroscopy; NS, not statistically significant; P, patients; $PaCO_2$, arterial partial pressure of CO_2; PE, phenylephrine; PM, perfusion modification; Perf. mod.; perfusion modification; PPG, photoplethysmography; R, right; ref, reference technique; rSO_2, regional tissue oxygen saturation; $S_{cap}O_2$, cerebral capillary oxygen saturation; $SctO_2$, scalp cerebral oxygen saturation; SD, standard deviation; SDS, source-detector separation; SE, supraorbital edge/ridge; SEM, standard error of the mean; $SjvO_2$, jugular venous oxygen saturation; sup, superior; TCD, transcranial doppler; temp, temporal/temporalis; TOI, Tissue Oxygenation Index; VA, vertebral artery.
NIRS manufacturers: EQUANOX, Nonin Medical; NIRO-300, NIRO 200NX, Hamamatsu Photonics; INVOS-3100, INVOS-3100A, INVOS-3100-SD, INVOS 4100, INVOS-5100B, INVOS-5100c, Somanetics; FORE-SIGHT (ELITE), CAS Medical Systems.

Author, Year, Sub-study	NIRS-device	SDS (cm)	Sensor location	Pop	n	Ref I	Ref E	PM	PM method	Methodology details	Results
**Tachtsidis 2008 [41]**	NIRO-300	5	Forehead, taking care to avoid the midline sinuses	H	10	TCD	LDF	Intra	Acetazolamide infusion	1 g over 2 min	TOI baseline: 70.1 +- 3.5%, TOI increase at acetazolamide injection: 1.8 +-2.4% (p<0.05)<br>CBFv baseline: 48.6+-28.1 cm/s. CBFv increase at acetazolamide injection: 21.8+-10.3 cm/s (p<0.01)<br>LDF: 'No statistically significant differences'<br>Correlation change TOI – change CBFv: r=0.77, p<0.01,
**Germon 1995-1 [42]**	INVOS 3100	3 and 4	Forehead, avoiding midline and m. temp	P	8	TCD	N/A	Intra	Hypercapnia	5% CO_2 inspiration for 3 min to stable $etCO_2$	rSO_2 to hypercapnia: 70.5 +-4 (mean +-SD)% to 74.6+-4 (mean +-SD)%, p=0.001<br>CBFv rest to hypercapnia: 37+13 (mean +-SD) to 80+-23 (mean +-SD) cm/s, p<0.01<br>Correlation rSO_2 – CBFv: r=0.229, p=0.621
**Germon 1995-3 [42]**	INVOS 3100	3 and 4	Forehead, avoiding midline and m. temp	P	8	TCD	N/A	Intra	Hypercapnia (during cuff inflation)	5% CO_2 inspiration for 3 min to stable $etCO_2$	rSO_2 scalp ischaemia versus hypercapnia+ scalp ischaemia: 62.6+-8 (mean +-SD)% to 64.5+-10 (mean +-SD)%, p=0.085<br>CBFv scalp ischaemia versus hypercapnia+ scalp ischaemia: 38+17 (mean +-SD) to 84+-24 (mean +-SD) cm/s, p<0.01<br>Correlation rSO_2 – CBFv: r=0.183, p=0.398
**Yoshitani 2002-3 [45]**	INVOS 4100	3.0 and 4.0	R forehead, caudal border 1 cm above eyebrow and medial edge at midline	P	19	TCD	N/A	Intra	Hypercapnia	Ventilatory rate changed from $PaCO_2$ 35-45 mmHg to 45-55 mmHg. MAP and HR unchanged	Change rSO_2 (INVOS) (mean+-SD): 15+-8%, TCD: 58+-29%
**Yoshitani 2002-4 [45]**	NIRO-300	4 and 5	R forehead, caudal border 1 cm above eyebrow and medial edge at midline	P	19	TCD	N/A	Intra	Hypercapnia	Ventilatory rate changed from $PaCO_2$ 35-45 mmHg to 45-55 mmHg. MAP and HR unchanged	Change TOI (NIRO) (mean+-SD): 8+-4%, TCD: 58+-29%
**Henson 1998 [46]**	INVOS 3100-SD	?	R forehead, caudal border at 1 cm above eyebrow and medial border at midline	H	30	$SjvO_2$	N/A	Intra	Hypercapnia	End-tidal forcing with $etCO_2$ of 2 mmHg above rest (baseline) to 7-10 mmHg above rest	rSO_2 (mean+-SD): 67+-8.3% to 71+-7.6%, $SjvO_2$ (mean+-SD): 64+-4.5% to 69+-5.5%
**Picton 2022 [43]**	INVOS 5100B	?	Right forehead	P	9	$PbrO_2$	N/A	Intra	Hypercapnia	Ventilatory FiO_2 and rate changed. FiO_2:0.3 and $PaCO_2$: 30 mmHg to FiO_2: 1.0 and $PaCO_2$: 40 mmHg	rSO_2 (med (IQR)): 68.0 (62.5-80.5)% to 83 (74.0-90.0), p=0.047<br>$PbrO_2$ (med (IQR)): 6.0 (4.0-11.3) to 22.5 (9.8-43.6) mmHg<br>Correlation rSO_2-$PbrO_2$: 0.50, p=0.036
**Sørensen 2015-1 [55]**	INVOS 4100	3 and 4	Below hairline and lat. on forehead	H	21	TCD, $S_{cap}O_2$	LDF	Intra	Hypocapnia	Hyperventilation. MAP unchanged, $PaCO_2$ decreased by -1.4 +-0.4 (95%CI)	Change NIRS (mean +- 95% CI): -3.4 +- 3.5 % (NS), TCD: -15 +- 9 cm/s, p<0.0001, LDF: 2.2+- 3.3 % (NS), $S_{cap}O_2$: -6.2 +-2.4, p<0.0001
**Sørensen 2015-8 [55]**	NIRO-200NX	3.5 and 4	Below hairline and lat. on forehead	H	21	TCD, $S_{cap}O_2$	LDF	Intra	Hypocapnia	Hyperventilation. MAP unchanged, $PaCO_2$ decreased by -1.4 +-0.4 (95%CI)	Change NIRS (mean +- 95% CI): -2.6+-4.2% (NS), TCD: -15 +- 9 cm/s, LDF: 2.2+- 3.3 % (NS), $S_{cap}O_2$: -6.2+-2.4%
**Grubhofer 1999-2 [47]**	INVOS 3100	?	On R or L forehead 2 cm above eyebrow and 2 cm from midline	H	15	N/A	N/A	Intra	Hypocapnia	Hyperventilation twice rest respiratory rate to $etCO_2$ < 20 mmHg	rSO_2: Rest to hypocapnia: 67.1% to 62.7%, p=0.025
**Yoshitani 2002-1 [45]**	INVOS 4100	3.0 and 4.0	R forehead, caudal border 1 cm above eyebrow and medial edge at midline	P	19	TCD	N/A	Intra	Hypocapnia	Ventilatory rate changed from $PaCO_2$ 35-45 mmHg to 25-35 mmHg. MAP and HR unchanged	rSO_2 $PaCO_2$ 25-35 mmHg (mean+-SD): 66+-8%. rSO_2 $PaCO_2$ 25-35: 60+-8%. Perc. change: -10+-5%, p<0.05<br>TCD $PaCO_2$ 25-35 mmHg (mean+-SD): 48+- 15 cm/s. TCD $PaCO_2$ 25-35: 34+-11 cm/s. Perc. change: -33+-14%, p<0.05
**Yoshitani 2002-2 [45]**	NIRO-300	4 and 5	R forehead, caudal border 1 cm above eyebrow and medial edge at midline	P	19	TCD	N/A	Intra	Hypocapnia	Ventilatory rate changed from $PaCO_2$ 35-45 mmHg to 25-35 mmHg. MAP and HR unchanged	TOI $PaCO_2$ 25-35 mmHg (mean+-SD): 66+-7% TOI $PaCO_2$ 25-35: 59+-7%. Perc. change: -11+-3%, p<0.05<br>TCD $PaCO_2$ 25-35 mmHg (mean+-SD): 48+- 15 cm/s. TCD $PaCO_2$ 25-35: 34+-11 cm/s. Perc. change: -33+-14%, p<0.05
**Canova 2011-4 [40]**	NIRO-300	5	High on L forehead excluding m. temp., lat. to midline to exclude sup sag sinus	H	22	TCD	PPG, NIRS on cheek, LDF	I	Hypocapnia	Hyperventilation with visual feedback to $etCO_2$ = 20 mmHg	No correlations available<br>ΔCBFv: -23.63+-14.46 % (p<0.01)<br>ΔPPG: 0.71+-2.07 AU (NS),<br>ΔtHb on cheek: 1.17+-3.73 muM (NS)<br>ΔLDF: 32.89+-43.30 % (NS), n=9, no correlations available<br>ΔrSO_2: -5.18+-3.04 %<br>ΔABP: -4.10 +-7.53%
**Canova 2011-5 [40]**	NIRO-300	5	High on L forehead excluding m. temp., lat. to midline to exclude sup sag sinus	H	24	TCD	PPG, NIRS on cheek, LDF	No	No	Valsalva maneuvre: Holding positive alveolar pressure of 40mmHg for 15s. Visual feedback + small leak prevented closing glottis	No correlations available<br>ΔCBFv: -6.75+-7.78 % (p<0.01)<br>ΔPPG: 3.74+-3.02 AU (p<0.01),<br>ΔtHb on cheek: 16.13+-8.81 muM (p<0.01)<br>ΔLDF: -16.71 +-37.00 % (NS), n=9, no correlations available<br>ΔrSO_2: -4.77 +-2.96 % (p<0.01)<br>ΔABP: 4.84 +-11.44 % (NS)

**Canova 2011-6 [40]**	NIRO-300	5	High on L forehead excluding m. temp., lat. to midline to exclude sup sag sinus	H	22	TCD	PPG, NIRS on cheek, LDF	No	No	Head-up tilt to 70 degrees for 5 min	No correlations available<br>ΔCBFv: -6.23+-6.10 % (p<0.05)<br>ΔPPG: -0.58+-3.30 AU (NS),<br>ΔtHb on cheek: -4.75 +-3.52 muM (NS)<br>ΔLDF: -27.47 +-13.24 % (p<0.01), n=9, no correlations available<br>ΔrSO_2: -3.19+-4.22 % (p<0.01)<br>ΔABP: 3.71+-19.52 % (NS)
**Al-Rawi 2001-1 [44]**	NIRO-300	5	High on forehead avoiding m. temp and sup. sagittal sinus	P	60	TCD	LDF	Intra	ICA clamping	ICA clamp 2 min after ECA clamp	Change TOI rest to ICA clamp (mean (SD)): -9.4 (7.1)% (subgroup of n=49 patients)<br>However, results from Table 1 (n=60), change TOI (mean (SD)): -7.8 (7.3) %<br>CBFv and LDF rest to ICA clamp (mean(SD)) not described in text, results from Table 1:<br>Change CBFv (mean (SD)): -24.3 (23.4) %<br>Change LDF (mean(SD)): -34.1 (20.9)%<br>Correlation CBFv-TOI: r=0.56, p<0.0001<br>Correlation LDF-TOI: r=0.13, not significant
**Samra 1999-1 [48]**	INVOS 3100A	3.0 and 4.0	Bilat. on forehead more than 3 cm from midline	P	34	N/A	N/A	Intra	ICA clamping	ICA clamp 5 min after ECA clamp (shunt in n=3 participants)	ScO_2 ECA-clamp to ICA-clamp (mean (SD)): 65.6 (8.3)% to 61.4 (9.6)%, not tested statistically
**Cho 1998-4 [49]**	INVOS 3100A	?	As much as possible over MCA territory, as close as possible to frontal hairline, avoiding midline	P	9	N/A	N/A	Intra	ICA unclamping	ICA unclamping 30-60 s after ECA unclamping	rSO_2 change cross-clamping to ICA unclamping: 4.92 (3.10)%
**Kato 2017-1 [50]**	ForeSight Elite	?	Frontal forehead	H	12	N/A	$S_{scalp}O_2$	Extra	Cuff inflation	Inflation to 20 mmHg above systolic BP - supine	Supine baseline (mean+-SD): 72+-5%, supine with scalp ischemia: 63+-5%, p<0.01<br>$S_{scalp}O_2$: ‘Loss of surface pulse oximetry plethysmography tracing’
**Kato 2017-2 [50]**	INVOS 5100c	?	Frontal forehead	H	12	N/A	$S_{scalp}O_2$	Extra	Cuff inflation	Inflation to 20 mmHg above systolic BP - supine	Supine baseline (mean+-SD) 74+-7%, supine with scalp ischemia: 61+-5%, p<0.01<br>$S_{scalp}O_2$: ‘Loss of surface pulse oximetry plethysmography tracing’
**Kato 2017-3 [50]**	ForeSight Elite	?	Frontal forehead	H	12	N/A	$S_{scalp}O_2$	Extra	Cuff inflation	Inflation to 20 mmHg above systolic BP - upright	Upright baseline (mean+-SD): 67+-4%, upright with scalp ischemia: 57+-6%, p<0.01<br>$S_{scalp}O_2$: ‘Loss of surface pulse oximetry plethysmography tracing’
**Kato 2017-4 [50]**	INVOS 5100c	?	Frontal forehead	H	12	N/A	$S_{scalp}O_2$	Extra	Cuff inflation	Inflation to 20 mmHg above systolic BP - upright	Upright baseline (mean+-SD): 71+-7%, upright with scalp ischemia: 56+-5%, p<0.01<br>$S_{scalp}O_2$: ‘Loss of surface pulse oximetry plethysmography tracing’
**Germon 1994-1 [51]**	INVOS 3100	?	R frontal area	H	8	N/A	N/A	Extra	Cuff inflation	Inflation above SE to 50 mmHg above systolic BP. ABP unchanged	Baseline versus tourniquet (mean (SD)): rSO_2 72 (6) to 59 (7)%, p<0.001
**Germon 1995-2 [51]**	INVOS 3100	3 and 4	Forehead, avoiding midline and m. temp	P	8	TCD	N/A	Extra	Cuff inflation	Inflation above SE to 200 mmHg. ABP unchanged	rSO_2 rest to scalp ischaemia: 70.5 +-4 (mean +-SD)% to 62.6+-8 (mean +-SD)%, not tested statistically<br>CBFv rest to scalp ischaemia: 37+13 (mean +-SD) to 38+-17 (mean +-SD) cm/s, not tested statistically
**Greenberg 2016-1 [52,53]**	INVOS 5100C	?	L side forehead	H	20	N/A	$S_{scalp}O_2$	Extra	Cuff inflation	Inflation to 20 mmHg above MAP	Change $SctO_2$ (median (IQR)): -15.1 (-17.6 to -12.6)%<br>Loss of $S_{scalp}O_2$ signal
**Greenberg 2016-2 [52,53]**	ForeSight Elite	?	L side forehead	H	20	N/A	$S_{scalp}O_2$	Extra	Cuff inflation	Inflation to 20 mmHg above MAP	Change $SctO_2$ (median (IQR)): -8.6 (-12.3 to -4.0)%<br>Loss of $S_{scalp}O_2$ signal
**Davie 2012-1 [54]**	Equanox	2.0 and 4.0	R side forehead	H	12	N/A	$S_{scalp}O_2$	Extra	Cuff inflation	Inflation above SE	Change $SctO_2$ Post 2 min inflation (mean (SD)): 6.6 (4.6)% decrease, Post 5 min inflation: 6.8 (6.0)% decrease<br>Loss of $S_{scalp}O_2$ signal
**Davie 2012-2 [54]**	ForeSight	1.5 and 5.0	R side forehead	H	12	N/A	$S_{scalp}O_2$	Extra	Cuff inflation	Inflation above SE	Change $SctO_2$ Post 2 min inflation (mean (SD)): 10.3 (5.2)% decrease, Post 5 min inflation: 11.8 (5.3)% decrease<br>Loss of $S_{scalp}O_2$ signal
**Davie 2012-3 [54]**	INVOS 5100C-PB	3.0 and 4.0	R side forehead	H	12	N/A	$S_{scalp}O_2$	Extra	Cuff inflation	Inflation above SE	Change $SctO_2$ Post 2 min inflation (mean (SD)): 13.9 (8.0)% decrease, Post 5 min inflation: 16.6 (9.6) % decrease<br>Loss of $S_{scalp}O_2$ signal
**Al-Rawi 2001-2 [44]**	NIRO-300	5	High on forehead avoiding m. temp and sup. saggital sinus	P	60	TCD	LDF	Extra	ECA clamping	2 min before ICA clamp	Change TOI (mean (SD)): -0.6 (2.1) %<br>Change LDF (mean (SD)): -43.7 (19.9)%<br>Change CBFv (mean (SD)): -2.6 (6.2) %
**Samra 1999-2 [48]**	INVOS 3100A	3.0 and 4.0	Bilat. on forehead more than 3 cm from midline	P	34	N/A	N/A	Extra	ECA clamping	5 min before ICA clamp	ScO_2 baseline to ECA-clamp (mean (SD)): 67.4 (8.5) to 65.6 (8.3)%, p=0.1187

**Cho 1998-3 [49]**	INVOS 3100A	?	As much as possible over MCA territory, as close as possible to frontal hairline, avoiding midline	P	9	N/A	N/A	Extra	ECA unclamping	30-60 s before ICA unclamping	rSO_2 change cross-clamping to ECA unclamping: 0.13 (0.39)%, N.S.
**Germon 1994-2 [51]**	INVOS 3100	?	R frontal area	H	12	N/A	N/A	Extra	Frontalis muscle contraction	1 min eyebrow raising	rSO_2 (mean(SD)) baseline to muscle contraction: 73 (7) % to 68 (6)%, p<0.001
**Ogoh 2014-1 [23]**	INVOS 4100	?	Above the SE and lat. on the forehead	H	7	TCD, Duplex (ICA), Duplex (VA)	LDF, Duplex (ECA)	Extra	Phenylephrine infusion	1.66 ± 0.08 μg/kg/min in 15 min.	Baseline: NIRS: ScO_2: 79.3 +-2.7% (mean+-SE). PE infusion: 63.9 +-1.9%, p=0.0005<br>Baseline ECA conductance (CBF/MAP) (mean+-SE): 1.86+-0.32 mL/min/mmHg to 0.94+-0.12 mL/min/mmHg, change -46.8+-3.9%, p=0.073<br>Correlations ScO_2 vs ECA conductance: Spearman's r = 0.64, p= 0.012<br>Baseline SkBF (LDF): 0.466+-0.097 (mean+-SE) PU. PE infusion: 0.318+-0.05, p=0.318<br>Correlations ScO_2 vs SkBF: Spearman' r = 0.81, p < 0.001<br>Baseline ICA conductance: 3.38+-0.32 (mean+-SE) mL/min/mmHg. PE infusion: 2.83+-0.24 mL/min/mmHg, change -14.8+-5.0%, p=0.165<br>No correlations calculated<br>Baseline VA conductance: 1.23+-0.19 (mean+-SE) mL/min/mmHg. PE infusion: 0.97+-0.16 mL/min/mmHg, change −21.8 ± 4.2%, p=0.318<br>No correlations calculated<br>Baseline MCA conductance index: 0.63+-0.06 (mean+-SE) cm/s/mmHg to 0.52+-0.05 cm/s/mmHg, change -16.7+-4.4, p=0.165<br>No correlation calculated
**Sørensen 2015-5 [55]**	INVOS 4100	3 and 4	Below hairline and lat. on forehead	H	7	TCD, $S_{cap}O_2$	LDF	Extra	Phenylephrine infusion	0.12 μg/kg/min	Change NIRS (mean +- 95% CI): -15.4+-6.4%, TCD: 8+-17 cm/s (NS), LDF: -6.5 +-6.3 , $S_{cap}O_2$: 1.2+-4.3 (NS)
**Sørensen 2015-12 [55]**	NIRO-200NX	3.5 and 4	Below hairline and lat. on forehead	H	?	TCD, $S_{cap}O_2$	LDF	Extra	Phenylephrine infusion	0.12 μg/kg/min	Change NIRS (mean +- 95% CI): -7.5+-7.1%, TCD: 8+-17 cm/s (NS), LDF: -6.5 +-6.3, $S_{cap}O_2$: 1.2+-4.3 (NS)
**Moerman 2021-2 [60]**	NIRO-200NX	?	L and R forehead	P	20	N/A	N/A	Extra	Phenylephrine infusion	100 μg whenever clinically indicated	TOI pre-PE versus post-PE (mean (95% CI)): -0.47 (-0.67-0.30)%, p < 0.001
**Sørensen 2015-2 [55]**	INVOS 4100	3 and 4	Below hairline and lat. on forehead	H	21	TCD, $S_{cap}O_2$	LDF	Extra	Whole body heating	Heating blanket around body and head until skin temp. ~1.5 °C above baseline	Change NIRS (mean +- 95% CI): 4.4+-3.6 %, TCD: -3+-9 cm/s (NS), LDF: 3.8+-3.3%, $S_{cap}O_2$: 0.7+-2.4% (NS)
**Sørensen 2015-9 [55]**	NIRO-200NX	3.5 and 4	Below hairline and lat. on forehead	H	21	TCD, $S_{cap}O_2$	LDF	Extra	Whole body heating	Heating blanket around body and head until skin temp. ~1.5 °C above baseline	Change NIRS (mean +- 95% CI): 0.3+-4.3% (NS), TCD: -3+-9 cm/s(NS), LDF: 3.8+-3.3%, $S_{cap}O_2$: 0.7+-2.4% (NS)
**Sørensen 2015-3 [55]**	INVOS 4100	3 and 4	Below hairline and lat. on forehead	H	15	TCD, $S_{cap}O_2$	LDF	No	No	Noradrenaline infusion: 0.15 μg/kg/min	Change NIRS (mean +- 95% CI): -6.0+-4.1 %, TCD: 0+-11 (NS), LDF: -2.2+-3.8 (NS), $S_{cap}O_2$: -0.5+-2.8 % (NS)
**Sørensen 2015-10 [55]**	NIRO-200NX	3.5 and 4	Below hairline and lat. on forehead	H	?	TCD, $S_{cap}O_2$	LDF	No	No	Noradrenaline infusion: 0.15 μg/kg/min	Change NIRS (mean +- 95% CI): -0.4 +-5.1%, TCD: 0+-11 (NS), LDF: -2.2+-3.8 (NS), $S_{cap}O_2$: -0.5+-2.8 % (NS)
**Sørensen 2015-4 [55]**	INVOS 4100	3 and 4	Below hairline and lat. on forehead	H	15	TCD, $S_{cap}O_2$	LDF	No	No	Noradrenaline infusion: 0.15 μg/kg/min. $etCO_2$ stabilisation by voluntary tidal volume restriction	Change NIRS (mean +- 95% CI): -4.3+-4.1%, TCD: 8+-11 (NS), LDF: -1.7+-3.8% (NS), $S_{cap}O_2$: 1.8+-2.8 (NS)
**Sørensen 2015-11 [55]**	NIRO-200NX	3.5 and 4	Below hairline and lat. on forehead	H	?	TCD, $S_{cap}O_2$	LDF	No	No	Noradrenaline infusion: 0.15 μg/kg/min. $etCO_2$ stabilisation by voluntary tidal volume restriction	Change NIRS (mean +- 95% CI): 0.3+-5.1% (NS), TCD: 8+-11 (NS), LDF: -1.7+-3.8% (NS), $S_{cap}O_2$: 1.8+-2.8 (NS)
**Sørensen 2015-6 [55]**	INVOS 4100	3 and 4	Below hairline and lat. on forehead	H	7	TCD, $S_{cap}O_2$	LDF	No	No	40° head-up tilt for 10 min	Change NIRS (mean +- 95% CI): -5.3+-6.3 % (NS), TCD: -6+-17 cm/s (NS), LDF: -0.4+-6.3 % (NS), $S_{cap}O_2$: -1.3+-4.3% (NS)
**Sørensen 2015-13 [55]**	NIRO-200NX	3.5 and 4	Below hairline and lat. on forehead	H	?	TCD, $S_{cap}O_2$	LDF	No	No	40° head-up tilt for 10 min	Change NIRS (mean +- 95% CI):-4.3+-7.1% (NS), TCD: -6+-17 cm/s (NS), LDF: -0.4+-6.3 % (NS), $S_{cap}O_2$: -1.3+-4.3% (NS)
**Sørensen 2015-7 [55]**	INVOS 4100	3 and 4	Below hairline and lat. on forehead	H	7	TCD, $S_{cap}O_2$	LDF	No	No	40° head-up tilt for 12 min. Concurrent PE infusion 0.12 μg/kg/min	Change NIRS (mean +- 95% CI): -15.1 +-6.3%, TCD: 3+-17 cm/s (NS), LDF: -2.5+-6.3% (NS), $S_{cap}O_2$: 0.8+-4.7% (NS)
**Sørensen 2015-14 [55]**	NIRO-200NX	3.5 and 4	Below hairline and lat. on forehead	H	?	TCD, $S_{cap}O_2$	LDF	No	No	40° head-up tilt for 12 min. Concurrent PE infusion 0.12 μg/kg/min	Change NIRS (mean +- 95% CI): -8.0+-7.0, TCD: 3+-17 cm/s (NS), LDF: -2.5+-6.3% (NS), $S_{cap}O_2$: 0.8+-4.7% (NS)
