# Supplementary material for: The Influence of Extracerebral Tissue on Continuous Wave Near-Infrared Spectroscopy in Adults: A Systematic Review of In Vivo Studies
Source: J Clin Med. 2023 Apr 8;12(8):2776. doi: 10.3390/jcm12082776 (PMC10146120; doi:10.3390/jcm12082776)
Supplement: Supplementary file 1 [file jcm-12-02776-s001.zip › Table S4.pdf]

Table S4: Critical appraisal classification results

Table S4: Results of the classification of the sub-studies studying haemoglobin (Hb) concentrations and tissue oxygen saturation indices (rSO_2) per QUADAS-2 domain. ECA, external carotid artery; fMRI, functional magnetic resonance imaging; ICA, internal carotid artery; LDF, laser doppler flowmetry; N/A, not applicable; NIRS; duplex, duplex ultrasonography; QUADAS-2, quality assessment of diagnostic accuracy studies second revised version; $S_{cap}O_2$, cerebral capillary oxygen saturation; SC-NIRS, short-channel (short source-detector distance); TCD, transcranial doppler; VA, vertebral artery.

**Author**	**Participant selection**	**Index test**	**Reference intracerebral**	**Reference extracerebral**	**Perfusion modification**	**Flow and timing**	**Participant selection – Applicability**	**Index test – Applicability**	**Reference intracerebral – Applicability**	**Reference extracerebral – Applicability**
**Haemoglobin (Hb) studies**										
**Cho 1998-1 [49]**	Unclear	Low	N/A	N/A	Low	High	High	Low	N/A	N/A
**Cho 1998-2** [49]	Unclear	Low	N/A	N/A	Low	High	High	Low	N/A	N/A
**Canova 2011-1 [40]**	Unclear	Low	Low	Low	Low	Low	Low	Low	Low	Low
**Canova 2011-2 [40]**	Unclear	Low	Low	Low	N/A	Low	Low	Low	Low	Low
**Canova 2011-3 [40]**	Unclear	Low	Low	Low	N/A	Low	Low	Low	Low	Low
**Funane 2015-1 [36]**	Unclear	High	Low	Unclear	N/A	Unclear	Low	Low	Low	Low
**Funane 2015-2 [36]**	Unclear	High	Low	Unclear	N/A	Unclear	Low	Low	Low	Low
**Funane 2015-3 [36]**	Unclear	High	Low	Unclear	N/A	Unclear	Low	Low	Low	Low
**Germon 1998-1 [57]**	Unclear	Low	Low	N/A	Low	High	Low	Low	Low	N/A
**Germon 1998-2 [57]**	Unclear	Low	Low	N/A	Low	High	Low	Low	Low	N/A
**Germon 1998-3 [57]**	Unclear	Low	N/A	N/A	Low	High	Low	Low	N/A	N/A
**Germon 1998-4 [57]**	Unclear	Low	N/A	N/A	Low	High	Low	Low	N/A	N/A
**Germon 1999-1 [56]**	Unclear	Low	Low	N/A	Low	Unclear	Low	Low	Low	N/A
**Germon 1999-2 [56]**	Unclear	Low	Low	N/A	Low	Unclear	Low	Low	Low	N/A
**Germon 1999-3 [56]**	Unclear	Low	Low	N/A	Low	Unclear	Low	Low	Low	N/A
**Germon 1999-4 [56]**	Unclear	Low	Low	N/A	Low	Unclear	Low	Low	Low	N/A
**Germon 1999-5 [56]**	Unclear	Low	Low	N/A	Low	Unclear	Low	Low	Low	N/A
**Germon 1999-6 [56]**	Unclear	Low	Low	N/A	Low	Unclear	Low	Low	Low	N/A

**Germon 1999-7 [56]**	Unclear	Low	Low	N/A	Low	Unclear	Low	Low	Low	N/A
**Germon 1999-8 [56]**	Unclear	Low	Low	N/A	Low	Unclear	Low	Low	Low	N/A
**Germon 1999-9 [56]**	Unclear	Low	N/A	N/A	Low	Unclear	Low	Low	N/A	N/A
**Germon 1999-10 [56]**	Unclear	Low	N/A	N/A	Low	Unclear	Low	Low	N/A	N/A
**Germon 1999-11 [56]**	Unclear	Low	N/A	N/A	Low	Unclear	Low	Low	N/A	N/A
**Germon 1999-12 [56]**	Unclear	Low	N/A	N/A	Low	Unclear	Low	Low	N/A	N/A
**Germon 1999-13 [56]**	Unclear	Low	N/A	N/A	Low	Unclear	Low	Low	N/A	N/A
**Germon 1999-14 [56]**	Unclear	Low	N/A	N/A	Low	Unclear	Low	Low	N/A	N/A
**Germon 1999-15 [56]**	Unclear	Low	N/A	N/A	Low	Unclear	Low	Low	N/A	N/A
**Germon 1999-16 [56]**	Unclear	Low	N/A	N/A	Low	Unclear	Low	Low	N/A	N/A
**Grubhofer 1999-1** [47]	Unclear	Low	N/A	N/A	Low	Unclear	Low	Low	N/A	N/A
**Haeussinger 2014 [37]**	Low	Low	Low	Low	N/A	High	Low	Low	Low	Low
**Heinzel 2013 [39]**	Low	Low	Low	Low	Low	High	Low	Low	Low	Low
**Hirasawa 2015-1 [33]**	Unclear	Low	N/A	Low	Low	Low	Low	Low	N/A	Low
**Hirasawa 2015-2 [33]**	Unclear	Low	N/A	Low	Low	Low	Low	Low	N/A	Low
**Hirasawa 2015-3 [33]**	Unclear	Low	N/A	Low	Low	Low	Low	Low	N/A	Low
**Hirasawa 2015-4 [33]**	Unclear	Low	N/A	Low	Low	Low	Low	Low	N/A	Low
**Hirasawa 2015-5 [33]**	Unclear	Low	N/A	Low	Low	Low	Low	Low	N/A	Low
**Hirasawa 2015-6 [33]**	Unclear	Low	N/A	Low	Low	Low	Low	Low	N/A	Low
**Hirasawa 2015-7 [33]**	Unclear	Low	N/A	Low	Unclear	Low	Low	Low	N/A	Low
**Hirasawa 2015-8 [33]**	Unclear	Low	N/A	Low	Unclear	Low	Low	Low	N/A	Low
**Hirasawa 2015-9 [33]**	Unclear	Low	N/A	Low	Unclear	Low	Low	Low	N/A	Low
**Hirasawa 2016-1 [59]**	Unclear	Low	N/A	Low	Low	Unclear	Low	Low	N/A	Low
**Hirasawa 2016-2 [59]**	Unclear	Unclear	N/A	Low	Low	Unclear	Low	High	N/A	Low
**Holzschuh 1997 [29]**	Unclear	Low	Low	N/A	Low	Unclear	High	Low	Low	N/A
**Kirkpatrick 1998-1 [32]**	High	High	Low	Low	Low	Unclear	High	High	Low	Low
**Kirkpatrick 1998-2 [32]**	High	High	Low	Low	Low	Unclear	High	High	Low	Low
**Lam 1997-1 [83]**	Unclear	Unclear	High	High	Low	High	High	Unclear	Low	Low

**Lam 1997-2 [83]**	Low	Unclear	High	High	Low	Unclear	High	Unclear	Low	Low
**Lam 1997-3 [83]**	Unclear	Unclear	High	High	Low	High	High	Unclear	Low	Low
**Lam 1997-4 [83]**	Low	Unclear	High	High	Low	Unclear	High	Unclear	Low	Low
**Moerman 2021-1 [61]**	Low	Low	N/A	N/A	Low	High	Low	Low	N/A	N/A
**Sato 2013-1 [38]**	High	High	Low	Low	N/A	High	Low	Low	Low	fMRI: Low<br>LDF: High
**Sato 2013-2 [38]**	High	High	Low	Low	N/A	High	Low	Low	Low	fMRI: Low<br>LDF: High
**Schecklmann 2017 [34]**	High	Low	N/A	Low	High	High	Low	Low	N/A	Low
**Smielewski 1995 [31]**	Low	Low	Low	Low	Low	High	Low	Low	Low	Low
**Smielewski 1997 [78]**	Unclear	High	N/A	Low	Unclear	High	High	Low	N/A	Low
**Smielewski 1998 [30]**	Unclear	Unclear	Low	Low	Low	High	High	Low	Low	Low
**Steinbrink 2003 [80]**	High	High	Low	Unclear	N/A	Unclear	Low	Low	Low	Unclear
**Takahashi 2011 [35]**	High	High	N/A	SC-NIRS: Low<br>LDF: Low	Low	High	Low	Low	N/A	SC-NIRS: Low<br>LDF: Low
**Tateishi 1995 [58]**	Unclear	Low	Low	N/A	Low	High	High	Low	Low	N/A
**Toronov 2001 [81]**	Low	Low	Unclear	Unclear	N/A	High	Low	Low	Unclear	Unclear
**Totaro 1998 [27]**	Low	Low	Low	N/A	Low	High	Low	Low	Low	N/A
**Yang 2020-1 [28]**	Unclear	Low	Low	N/A	Low	High	Low	Low	Low	N/A
**Yang 2020-2 [28]**	Unclear	Low	Low	N/A	Low	High	Low	Low	Low	N/A
**Yang 2020-3 [28]**	Unclear	Low	Low	N/A	Low	High	Low	Low	Low	N/A
**Yang 2020-4 [28]**	Unclear	Low	Low	N/A	Low	High	Low	Low	Low	N/A
**Zarei 2019 [77]**	Unclear	Unclear	N/A	N/A	High	Unclear	Low	Unclear	N/A	N/A
**Tissue oxygen saturation indices (rSO$_2$) studies**										
**Al-Rawi 2001-1 [44]**	Unclear	Low	Low	Low	Low	Unclear	High	Low	Low	Low
**Al-Rawi 2001-2 [44]**	Unclear	Low	Low	Low	Low	Unclear	High	Low	Low	Low
**Cho 1998-3 [49]**	Unclear	Low	N/A	N/A	Low	High	High	Low	N/A	N/A
**Cho 1998-4 [49]**	Unclear	Low	N/A	N/A	Low	High	High	Low	N/A	N/A

**Canova 2011-4 [40]**	Unclear	Low	Low	Low	Low	Unclear	Low	Low	Low	Low
**Canova 2011-5 [40]**	Unclear	Low	Low	Low	N/A	Unclear	Low	Low	Low	Low
**Canova 2011-6 [40]**	Unclear	Low	Low	Low	N/A	Unclear	Low	Low	Low	Low
**Davie 2012-1 [54]**	Unclear	Low	N/A	Unclear	Low	Unclear	Low	Low	N/A	Low
**Davie 2012-2 [54]**	Unclear	Low	N/A	Unclear	Low	Unclear	Low	Low	N/A	Low
**Davie 2012-3 [54]**	Unclear	Low	N/A	Unclear	Low	Unclear	Low	Low	N/A	Low
**Duncan 1995-1 [82]**	Unclear	Low	N/A	N/A	Low	Unclear	High	Unclear	N/A	N/A
**Duncan 1995-2 [82]**	Unclear	Low	N/A	N/A	Low	Unclear	High	Unclear	N/A	N/A
**Germon 1994-1 [51]**	Unclear	Low	N/A	N/A	Low	High	Low	Low	N/A	N/A
**Germon 1994-2 [51]**	Unclear	Low	N/A	N/A	High	High	Low	Low	N/A	N/A
**Germon 1995-1 [42]**	Unclear	Low	Low	N/A	Low	Unclear	Low	Low	Low	N/A
**Germon 1995-2 [42]**	Unclear	Low	Low	N/A	Low	Unclear	Low	Low	Low	N/A
**Germon 1995-3 [42]**	Unclear	Low	Low	N/A	Low	Unclear	Low	Low	Low	N/A
**Greenberg 2016-1 [52,53]**	Low	Low	N/A	Unclear	Low	High	Low	Low	N/A	Low
**Greenberg 2016-2 [52,53]**	Low	Low	N/A	Unclear	Low	High	Low	Low	N/A	Low
**Grubhofer 1999-2 [47]**	Unclear	Low	N/A	N/A	Low	Unclear	Low	Low	N/A	N/A
**Henson 1998 [46]**	Unclear	Low	Low	N/A	Low	High	Low	Low	Low	N/A
**Kato 2017-1 [50]**	Low	Low	N/A	Unclear	Low	Low	Low	Low	N/A	Low
**Kato 2017-2 [50]**	Low	Low	N/A	Unclear	Low	Low	Low	Unclear	N/A	Low
**Kato 2017-3 [50]**	Low	Low	N/A	Unclear	Low	Low	Low	Unclear	N/A	Low
**Kato 2017-4 [50]**	Low	Low	N/A	Unclear	Low	Low	Low	Unclear	N/A	Low
**Moerman 2021-2 [60]**	Low	Low	N/A	N/A	Low	High	Low	Low	N/A	N/A
**Ogoh 2014 [23]**	Unclear	Low	Duplex ICA: Low TCD: Low Duplex VA: Low	Duplex ECA: Low LDF: Low	Low	High	Low	Low	Duplex ICA: Low TCD: Low Duplex VA: Low	Duplex ECA: Low LDF: Low
**Picton 2022 [43]**	Low	Low	Low	N/A	Low	Unclear	High	Low	Low	N/A

**Samra 1999-1 [48]**	Unclear	Low	N/A	N/A	Low	High	High	Low	N/A	N/A
**Samra 1999-2 [48]**	Unclear	Low	N/A	N/A	Low	High	High	Low	N/A	N/A
**Sørensen 2015-1 [55]**	Unclear	Low	TCD: Low<br>$S_{cap}O_2$: Low	Low	Low	Unclear	Low	Low	Low	Low
**Sørensen 2015-2 [55]**	Unclear	Low	TCD: Low<br>$S_{cap}O_2$: Low	Low	Low	Unclear	Low	Low	Low	Low
**Sørensen 2015-3 [55]**	Unclear	Low	TCD: Low<br>$S_{cap}O_2$: Low	Low	High	Unclear	Low	Low	Low	Low
**Sørensen 2015-4 [55]**	Unclear	Low	TCD: Low<br>$S_{cap}O_2$: Low	Low	High	Unclear	Low	Low	Low	Low
**Sørensen 2015-5 [55]**	Unclear	Low	TCD: Low<br>$S_{cap}O_2$: Low	Low	Low	Unclear	Low	Low	Low	Low
**Sørensen 2015-6 [55]**	Unclear	Low	TCD: Low<br>$S_{cap}O_2$: Low	Low	High	Unclear	Low	Low	Low	Low
**Sørensen 2015-7 [55]**	Unclear	Low	TCD: Low<br>$S_{cap}O_2$: Low	Low	High	Unclear	Low	Low	Low	Low
**Sørensen 2015-8 [55]**	High	Low	TCD: Low<br>$S_{cap}O_2$: Low	Low	Low	Unclear	Low	Low	Low	Low
**Sørensen 2015-9 [55]**	High	Low	TCD: Low<br>$S_{cap}O_2$: Low	Low	Low	Unclear	Low	Low	Low	Low
**Sørensen 2015-10 [55]**	High	Low	TCD: Low<br>$S_{cap}O_2$: Low	Low	High	Unclear	Low	Low	Low	Low
**Sørensen 2015-11 [55]**	High	Low	TCD: Low<br>$S_{cap}O_2$: Low	Low	High	Unclear	Low	Low	Low	Low
**Sørensen 2015-12 [55]**	High	Low	TCD: Low<br>$S_{cap}O_2$: Low	Low	Low	Unclear	Low	Low	Low	Low
**Sørensen 2015-13 [55]**	High	Low	TCD: Low<br>$S_{cap}O_2$: Low	Low	High	Unclear	Low	Low	Low	Low
**Sørensen 2015-14 [55]**	High	Low	TCD: Low<br>$S_{cap}O_2$: Low	Low	High	Unclear	Low	Low	Low	Low

**Tachtsidis 2008 [41]**	Unclear	Low	Low	Low	Low	High	Low	Low	Low	Low
**Takeda 2000 [79]**	Unclear	Low	Low	N/A	Unclear	High	High	Unclear	Low	N/A
**Yoshitani 2002-1 [45]**	Low	Low	Low	N/A	Low	Unclear	Low	Low	Low	N/A
**Yoshitani 2002-2 [45]**	Low	Low	Low	N/A	Low	Unclear	Low	Low	Low	N/A
**Yoshitani 2002-3 [45]**	Low	Low	Low	N/A	Low	Unclear	Low	Low	Low	N/A
**Yoshitani 2002-4 [45]**	Low	Low	Low	N/A	Low	Unclear	Low	Low	Low	N/A

References

1. Cho, H.; Nemoto, E.M.; Yonas, H.; Balzer, J.; Sclabassi, R.J. Cerebral Monitoring by Means of Oximetry and Somatosensory Evoked Potentials during Carotid Endarterectomy. *J Neurosurg* **1998**, *89*, 533–538, doi:10.3171/jns.1998.89.4.0533.

2. Canova, D.; Roatta, S.; Bosone, D.; Micieli, G. Inconsistent Detection of Changes in Cerebral Blood Volume by near Infrared Spectroscopy in Standard Clinical Tests. *J Appl Physiol* **2011**, *110*, 1646–1655, doi:10.1152/japplphysiol.00003.2011.

3. Funane, T.; Sato, H.; Yahata, N.; Takizawa, R.; Nishimura, Y.; Kinoshita, A.; Katura, T.; Atsumori, H.; Fukuda, M.; Kasai, K.; et al. Concurrent FNIRS-FMRI Measurement to Validate a Method for Separating Deep and Shallow FNIRS Signals by Using Multidistance Optodes. *Neurophotonics* **2015**, *2*, 015003, doi:10.1117/1.nph.2.1.015003.

4. Germon, T.J.; Evans, P.D.; Manara, A.R.; Barnett, N.J.; Wall, P.; Nelson, R.J. Sensitivity of near Infrared Spectroscopy to Cerebral and Extra-Cerebral Oxygenation Changes Is Determined Dy Emitter-Detector Separation. *J Clin Monit Comput* **1998**, *14*, 353–360, doi:10.1023/A:1009957032554.

5. Germon, T.J.; Evans, P.D.; Barnett, N.J.; Wall, P.; Manara, A.R.; Nelson, R.J. Cerebral near Infrared Spectroscopy: Emitter-Detector Separation Must Be Increased. *Br J Anaesth* **1999**, *82*, 831–837, doi:10.1093/bja/82.6.831.

6. Grubhofer, G.; Tonninger, W.; Keznickl, P.; Skyllouriotis, P.; Ehrlich, M.; Hiesmayr, M.; Lassnigg, A. A Comparison of the Monitors INVOS 3100 and NIRO 500 in Detecting Changes in Cerebral Oxygenation. *Acta Anaesthesiol Scand* **1999**, *43*, 470–475, doi:10.1034/j.1399-6576.1999.430417.x.

7. Haeussinger, F.B.; Dresler, T.; Heinzel, S.; Schecklmann, M.; Fallgatter, A.J.; Ehlis, A.C. Reconstructing Functional Near-Infrared Spectroscopy (FNIRS) Signals Impaired by Extra-Cranial Confounds: An Easy-to-Use Filter Method. *Neuroimage* **2014**, *95*, 69–79, doi:10.1016/j.neuroimage.2014.02.035.

8. Heinzel, S.; Haeussinger, F.B.; Hahn, T.; Ehlis, A.C.; Plichta, M.M.; Fallgatter, A.J. Variability of (Functional) Hemodynamics as Measured with Simultaneous FNIRS and FMRI during Intertemporal Choice. *Neuroimage* **2013**, *71*, 125–134, doi:10.1016/j.neuroimage.2012.12.074.

9. Hirasawa, A.; Yanagisawa, S.; Tanaka, N.; Funane, T.; Kiguchi, M.; Sørensen, H.; Secher, N.H.; Ogoh, S. Influence of Skin Blood Flow and Source-Detector Distance on near-Infrared Spectroscopy-Determined Cerebral Oxygenation in Humans. *Clin Physiol Funct Imaging* **2015**, *35*, 237–244, doi:10.1111/cpf.12156.

10. Hirasawa, A.; Kaneko, T.; Tanaka, N.; Funane, T.; Kiguchi, M.; Sørensen, H.; Secher, N.H.; Ogoh, S. Near-Infrared Spectroscopy Determined Cerebral Oxygenation with Eliminated Skin Blood Flow in Young Males. *J Clin Monit Comput* **2016**, *30*, 243–250, doi:10.1007/s10877-015-9709-4.

11. Holzschuh, M.; Woertgen, C.; Metz, C.; Brawanski, A. Comparison of Changes in Cerebral Blood Flow and Cerebral Oxygen Saturation Measured by Near Infrared Spectroscopy (NIRS) after Acetazolamide. *Acta Neurochir (Wien)* **1997**, *139*, 58–62, doi:10.1007/BF01850869.

12. Kirkpatrick, P.J.; Lam, J.; Al-Rawi, P.; Smielewski, P.; Czosnyka, M. Defining Thresholds for Critical Ischemia by Using Near-Infrared Spectroscopy in the Adult Brain. *J Neurosurg* **1998**, *89*, 389–394, doi:10.3171/jns.1998.89.3.0389.

13. Lam, J.M.K.K.; Smielewski, P.; Al-Rawi, P.; Griffiths, P.; Pickard, J.D.; Kirkpatrick, P.J. Internal and External Carotid Contributions to Near-Infrared Spectroscopy during Carotid Endarterectomy. *Stroke* **1997**, *28*, 906–911, doi:10.1161/01.STR.28.5.906.

14. Moerman, A.T.; Vandenheuvel, M.; Tuybens, P.J.; Van Gompel, C.; De Hert, S.G. Incongruous Effect of Phenylephrine on Changes in Cerebral Blood Volume Measured by Near-Infrared Spectroscopy (NIRS) Indicating Extracranial Contamination. *J Clin Monit Comput* **2021**, doi:10.1007/s10877-021-00702-3.

15. Sato, H.; Yahata, N.; Funane, T.; Takizawa, R.; Katura, T.; Atsumori, H.; Nishimura, Y.; Kinoshita, A.; Kiguchi, M.; Koizumi, H.; et al. A NIRS-FMRI Investigation of Prefrontal Cortex Activity during a Working Memory Task. *Neuroimage* **2013**, *83*, 158–173, doi:10.1016/j.neuroimage.2013.06.043.

16. Schecklmann, M.; Mann, A.; Langguth, B.; Ehlis, A.C.; Fallgatter, A.J.; Haeussinger, F.B. The Temporal Muscle of the Head Can Cause Artifacts in Optical Imaging Studies with Functional Near-Infrared Spectroscopy. *Front Hum Neurosci* **2017**, *11*, 456, doi:10.3389/fnhum.2017.00456.

17. Smielewski, P.; Kirkpatrick, P.; Minhas, P.; Pickard, J.D.; Czosnyka, M. Can Cerebrovascular Reactivity Be Measured with Near-Infrared Spectroscopy? *Stroke* **1995**, *26*, 2285–2292, doi:10.1161/01.STR.26.12.2285.

18. Smielewski, P.; Czosnyka, M.; Pickard, J.D.; Kirkpatrick, P. Clinical Evaluation of Near-Infrared Spectroscopy for Testing Cerebrovascular Reactivity in Patients with Carotid Artery Disease. *Stroke* **1997**, *28*, 331–338, doi:10.1161/01.STR.28.2.331.

19. Smielewski, P.; Czosnyka, M.; Pickard, J.D.; Kirkpatrick, P. Assessment of Cerebrovascular Reactivity in Patients with Carotid Artery Disease Using Near-Infrared Spectroscopy. *Acta Neurochir Suppl* **1998**, *1998*, 263–265, doi:10.1007/978-3-7091-6475-4_76.

20. Steinbrink, J.M.; Kempf, F.; Schreiber, S.; Uludag, K.; Kohl, M.; Valdueza, J.M.; Villringer, A.; Obrig, H. Functional Brain Imaging by CW-NIRS Coregistered by Blood Flow Monitors. In Proceedings of the Photon Migration and Diffuse-Light Imaging; 2003; Vol. 5138.

21. Takahashi, T.; Takikawa, Y.; Kawagoe, R.; Shibuya, S.; Iwano, T.; Kitazawa, S. Influence of Skin Blood Flow on Near-Infrared Spectroscopy Signals Measured on the Forehead during a Verbal Fluency Task. *Neuroimage* **2011**, *57*, 991–1002, doi:10.1016/j.neuroimage.2011.05.012.

22. Tateishi, A.; Maekawa, T.; Soejima, Y.; Sadamitsu, D.; Yamamoto, M.; Matsushita, M.; Nakashima, K. Qualitative Comparison of Carbon Dioxide-Induced Change in Cerebral near- Infrared Spectroscopy versus Jugular Venous Oxygen Saturation in Adults with Acute Brain Disease. *Crit Care Med* **1995**, *23*, 1734–1738, doi:10.1097/00003246-199510000-00019.

23. Toronov, V.Y.; Webb, A.; Choi, J.H.; Wolf, M.; Gratton, E.; Hueber, D.M. Simultaneous Functional Magnetic Resonance and Near-Infrared Imaging of Adult Human Brain. In Proceedings of the Optical Tomography and Spectroscopy of Tissue IV; SPIE, June 29 2001; Vol. 4250, pp. 380–382.

24. Totaro, R.; Barattelli, G.; Quaresima, V.; Carolei, A.; Ferrari, M. Evaluation of Potential Factors Affecting the Measurement of Cerebrovascular Reactivity by Near-Infrared Spectroscopy. *Clin Sci* **1998**, *95*, 497–504, doi:10.1042/CS19980122.

25. Yang, H.C.; Liang, Z.; Vike, N.L.; Lee, T.; Rispoli, J. V.; Nauman, E.A.; Talavage, T.M.; Tong, Y. Characterizing Near-Infrared Spectroscopy Signal under Hypercapnia. *J Biophotonics* **2020**, *13*, e202000173, doi:10.1002/jbio.202000173.

26. Zarei, M.; Ansari, M.A.; Zare, K. The Temporal Confounding Effects of Extra-Cerebral Contamination Factors on the Hemodynamic Signal Measured by Functional near-Infrared Spectroscopy. *J Lasers Med Sci* **2019**, *10*, S73–S81, doi:10.15171/jlms.2019.S14.

27. Al-Rawi, P.G.; Smielewski, P.; Kirkpatrick, P.J. Evaluation of a Near-Infrared Spectrometer (NIRO 300) for the Detection of Intracranial Oxygenation Changes in the Adult Head. *Stroke* **2001**, *32*, 2492–2499, doi:10.1161/hs1101.098356.

28. Davie, S.N.; Grocott, H.P. Impact of Extracranial Contamination on Regional Cerebral Oxygen Saturation: A Comparison of Three Cerebral Oximetry Technologies. *Anesthesiology* **2012**, *116*, 834–840, doi:10.1097/ALN.0b013e31824c00d7.

29. Duncan, L.A.; Ruckley, C.V.; Wildsmith, J.A. Cerebral Oximetry: A Useful Monitor during Carotid Artery Surgery. *Anaesthesia* **1995**, *50*, 1041–1045, doi:10.1111/j.1365-2044.1995.tb05947.x.

30. Germon, T.J.; Kane, N.M.; Manara, A.R.; Nelson, R.J. Near-Infrared Spectroscopy in Adults: Effects of Extracranial Ischaemia and Intracranial Hypoxia on Estimation of Cerebral Oxygenation. *Br J Anaesth* **1994**, *73*, 503–506, doi:10.1093/bja/73.4.503.

31. Germon, T.J.; Young, A.E.R.; Manara, A.R.; Nelson, R.J. Extracerebral Absorption of near Infrared Light Influences the Detection of Increased Cerebral Oxygenation Monitored by near Infrared Spectroscopy. *J Neurol Neurosurg Psychiatry* **1995**, *58*, 477–479, doi:10.1136/jnnp.58.4.477.

32. Greenberg, S.; Murphy, G.; Shear, T.; Patel, A.; Simpson, A.; Szokol, J.; Avram, M.J.; Vender, J. Extracranial Contamination in the INVOS 5100C versus the FORE-SIGHT ELITE Cerebral Oximeter: A Prospective Observational Crossover Study in Volunteers. *Canadian Journal of Anesthesia* **2016**, *63*, 24–30, doi:10.1007/s12630-015-0451-7.

33. Greenberg, S.; Murphy, G.; Shear, T.; Patel, A.; Simpson, A.; Szokol, J.; Avram, M.J.; Vender, J. Erratum to: Extracranial Contamination in the INVOS 5100C versus the FORE-SIGHT ELITE Cerebral Oximeter: A Prospective Observational Crossover Study in Volunteers. *Canadian Journal of Anesthesia* 2016, *63*.

34. Henson, L.C.; Temp, J.A.; Ward, D.S. Accuracy of a Cerebral Oximeter in Healthy Volunteers under Conditions of Isocapnic Hypoxia. *Anesthesiology* **1998**, *88*, 58–65, doi:10.1097/00000542-199801000-00011.

35. Kato, S.; Yoshitani, K.; Kubota, Y.; Inatomi, Y.; Ohnishi, Y. Effect of Posture and Extracranial Contamination on Results of Cerebral Oximetry by Near-Infrared Spectroscopy. *J Anesth* **2017**, *31*, 103–110, doi:10.1007/s00540-016-2275-1.

36. Ogoh, S.; Sato, K.; Okazaki, K.; Miyamoto, T.; Secher, F.; Sørensen, H.; Rasmussen, P.; Secher, N.H. A Decrease in Spatially Resolved Near-Infrared Spectroscopy-Determined Frontal Lobe Tissue Oxygenation by Phenylephrine Reflects Reduced Skin Blood Flow. *Anesth Analg* **2014**, *118*, 823–829, doi:10.1213/ANE.0000000000000145.

37. Picton, P.; Vlisides, P.E.; Teig, M.K.; Heth, J.A.; Orringer, D.; Brooks, J.; McKinney, A.; Mentz, G.; Mashour, G.A. Correlation between Brain Tissue Oxygen Tension and Regional Cerebral Oximetry in Uninjured Human Brain under Conditions of Changing Ventilation Strategy. *J Clin Monit Comput* **2022**, doi:10.1007/s10877-022-00821-5.

38. Samra, S.K.; Stanley, J.C.; Zelenock, G.B.; Dorje, P. An Assessment of Contributions Made by Extracranial Tissues during Cerebral Oximetry. *J Neurosurg Anesthesiol* **1999**, *11*, 1–5, doi:10.1097/00008506-199901000-00001.

39. Sørensen, H.; Rasmussen, P.; Siebenmann, C.; Zaar, M.; Hvidtfeldt, M.; Ogoh, S.; Sato, K.; Kohl-Bareis, M.; Secher, N.H.; Lundby, C. Extra-Cerebral Oxygenation Influence on near-Infrared-Spectroscopy-Determined Frontal Lobe Oxygenation in Healthy Volunteers: A Comparison between INVOS-4100 and NIRO-200NX. *Clin Physiol Funct Imaging* **2015**, *35*, 177–184, doi:10.1111/cpf.12142.

40. Tachtsidis, I.; Tisdall, M.; Delpy, D.T.; Smith, M.; Elwell, C.E. Measurement of Cerebral Tissue Oxygenation in Young Healthy Volunteers during Acetazolamide Provocation: A Transcranial Doppler and near-Infrared Spectroscopy Investigation. *Adv Exp Med Biol* **2008**, *614*, 389–396, doi:10.1007/978-0-387-74911-2_43.

41. Takeda, N.; Fujita, K.; Katayama, S.; Tamaki, N. Cerebral Oximetry for the Detection of Cerebral Ischemia during Temporary Carotid Artery Occlusion. *Neurol Med Chir (Tokyo)* **2000**, *40*, 557–563, doi:10.2176/nmc.40.557.

42. Yoshitani, K.; Kawaguchi, M.; Tatsumi, K.; Kitaguchi, K.; Furuya, H. A Comparison of the INVOS 4100 and the NIRO 300 Near-Infrared Spectrophotometers. *Anesth Analg* **2002**, *94*, 586–590, doi:10.1097/00000539-200203000-00020.
