# Supplementary material for: The Influence of Extracerebral Tissue on Continuous Wave Near-Infrared Spectroscopy in Adults: A Systematic Review of In Vivo Studies
Source: J Clin Med. 2023 Apr 8;12(8):2776. doi: 10.3390/jcm12082776 (PMC10146120; doi:10.3390/jcm12082776)
Supplement: Supplementary file 1 [file jcm-12-02776-s001.zip › Table S5.pdf]

# Table S5: Studies excluded post hoc based on study quality

Seven articles comprising 11 sub-studies were excluded post hoc due to poor study quality. These studies scored a high or unclear risk of bias or had high or unclear applicability concerns in more than 50% Quality Assessment of Diagnostic Accuracy Studies 2 (QUADAS-2) domains. Critical appraisal results are shown in Table S5.

Table S5: Critical appraisal results of post hoc excluded studies using the modified Quality Assessment of Diagnostic Accuracy Studies 2 (QUADAS-2) tool.
Studies were classified per regarding risk of bias or applicability concerns as 'Low', 'High', 'Unclear', 'Not applicable (N/A)'. N/A could only apply to the domains regarding Reference techniques or Perfusion modification.

| Author | Participant selection | Index test | Reference intracerebral | Reference extracerebral | Perfusion modification | Flow and timing | Subject selection – Applicability | Index test – Applicability | Reference intracerebral – Applicability | Reference extracerebral – Applicability |
|---|---|---|---|---|---|---|---|---|---|---|
| **Zarei 2019 [77]** | Unclear | Unclear | N/A | N/A | High | Unclear | Low | Unclear | N/A | N/A |
| **Smielewski 1997 [78]** | Unclear | High | N/A | Low | Unclear | High | High | Low | N/A | Low |
| **Takeda 2000 [79]** | Unclear | Low | Low | N/A | Unclear | High | High | Unclear | Low | N/A |
| **Steinbrink 2003 [80]** | High | High | Low | Unclear | N/A | Unclear | Low | Low | Low | Unclear |
| **Toronov 2001 [81]** | Low | Low | Unclear | Unclear | N/A | High | Low | Low | Unclear | Unclear |
| **Duncan 1995-1 [82]** | Unclear | Low | N/A | N/A | Low | Unclear | High | Unclear | N/A | N/A |
| **Duncan 1995-2 [82]** | Unclear | Low | N/A | N/A | Low | Unclear | High | Low | N/A | N/A |
| **Lam 1997-1 [83]** | Unclear | Unclear | High | High | Low | High | High | Unclear | Low | Low |
| **Lam 1997-2 [83]** | Low | Unclear | High | High | Low | Unclear | High | Unclear | Low | Low |
| **Lam 1997-3 [83]** | Unclear | Unclear | High | High | Low | High | High | Unclear | Low | Low |
| **Lam 1997-4 [83]** | Low | Unclear | High | High | Low | Unclear | High | Unclear | Low | Low |
