# Supplementary material for: The Influence of Extracerebral Tissue on Continuous Wave Near-Infrared Spectroscopy in Adults: A Systematic Review of In Vivo Studies
Source: J Clin Med. 2023 Apr 8;12(8):2776. doi: 10.3390/jcm12082776 (PMC10146120; doi:10.3390/jcm12082776)
Supplement: Supplementary file 1 [file jcm-12-02776-s001.zip › Table S6.pdf]

Table S6: Detailed study characteristics of studies excluded post-hoc

Seven articles comprising 11 sub-studies were excluded post-hoc due to poor study quality. These studies scored a high or unclear risk of bias or had high or unclear applicability concerns in more than 50% Quality Assessment of Diagnostic Accuracy Studies 2 (QUADAS-2) domains. Study characteristics are provided in Table S6.

Table S6: Study characteristics of post-hoc excluded studies.
ABP, arterial blood pressure; BOLD, blood-oxygen-level-dependent (signal); CBFv, cerebral blood flow velocity; cor, coronal; E/Extra, extracerebral; ECA, external carotid artery; fMRI, functional magnetic resonance spectroscopy; H, healthy participants; Hb, haemoglobin; HHb, deoxygenated Hb; I/intra, intracerebral; ICA, internal carotid artery; LDF, laser doppler flowmetry; m., muscle; N/A, not applicable; NIRS, near-infrared spectroscopy; OxyHb, oxygenated Hb; P, patients; Perf. mod, perfusion modification; Pop., population; SDS, source-detector separation; SE, supraorbital edge/ridge; TCD, transcranial doppler.
NIRS manufacturers: NIRO-500, Hamamatsu Photonics; INVOS 3100, INVOS 3100A, Somanetics; Frequency domain oximeter, ISS.

Author	NIRS-device	SDS (cm)	Sensor location	Pop.	n	Ref intra	Ref extra	I/E perf. mod.?	Perf. mod. performed	Perfusion modification description	Results
**Zarei 2019 [77]**	Custom made	3	L frontal pole (Fp1) and R frontal pole (Fp2)	H	6	N/A	N/A	E	Laser scalp blood flow stimulation	Scalp blood flow stimulation with 1550nm wavelength laser in middle Fz-Fpz (EEG 10-20) line. >3 cm away from NIRS optodes	No summary statistics described. Change upon SBFAP (scalp blood flow activation by photobiomodulation):<br>- OxyHb (left): 0.6,0.6,1.1,1.2,0.6,0.8 μM → mean change 0.82 μM<br>- OxyHb (right): 0.3,0.6,0.6,0.8,0.4,0.6 μM → mean change 0.55 μM<br>- HHb (left) : -0.5,-0.6,-1.0,-0.1,-0.5,-0.1 μM → mean change -0.47 μM<br>- HHb (right): -0.4,-0.5,-0.9,-0.5,-0.6,-0.2 μM → mean change -0.52 μM
**Smielewski 1997 [78]**	NIRO-500	6	Receiver 2 cm lateral to midline and 2 cm coronal to SE. Transmitter 6 cm toward hairline	P	69		LDF	E	Temporal artery compression	10 sec compressions of ipsilateral temporal artery	Ipsilateral: Change OxyHb of 1.85 μmol/L per 100% change in LDF (range, 0 to 4.9 μmol/L)<br>Contralateral: Change OxyHb of 1.56 μmol/L per 100% change in LDF (range, 0 to 5.4 μmol/L)
**Takeda 2000-1 [79]**	INVOS 3100A	3.0 and 4.0	Bilateral on forehead more than 3 cm from midline	P	9	N/A	N/A	Intra	ICA balloon occlusion	Occlusion confirmed by contrast medium stasis	rSO_2: 67.4,74.0,56.6,78.8,NA,62.0,76.4,67.4,69.8 → mean 69.1%<br>Change rSO_2 with balloon occlusion: -2.4, -2.4, -3.0, -4.2, NA, -4.2, -4.2, -0.6, -2.4 % (percentage point) → mean change -2.9 %
**Steinbrink 2003 [80]**	Custom made	?	Occipital lobes	H	10	TCD	LDF	No	No	Visual hemi-field stimulation with	No quantitative results:<br>OxyHb: Increase in OxyHb in maximum amplitude pixels reached a maximum 12 s

										annular checkerboard at 8 Hz	after visual hemifield stimulus onset. 2.5 μM increase (estimated from graph)<br>HHb: Decrease in HHb in maximum amplitude pixels reached a maximum 8 s after Vis Hemifield stimulus onset. 1.5 μM decrease (estimated from graph)<br>TCD of posterior cerebral artery reached a maximum 6 s after after visual hemifield stimulus onset. No units. ~ 2 AU increase (estimated from graph)<br>LDF: 'LDF measurements did not return a significant BV or BF effect (not shown)'<br>ABP: 'no systemic change in aBP across subjects'
**Toronov 2001 [81]**	Frequency domain oximeter	3	Covering area around C3	H	6	fMRI	fMRI	No	No	Light palm squeezing with right hand for 10 20-sec epochs	fMRI BOLD signal highly correlated with paradigm boxcar function in primary motor cortex.<br>Significant decrease of HHb during the palm squeezing in channels situated above primary motor cortex,<br>Significant increase of OxyHb during the palm squeezing in channels situated above primary motor cortex,<br>Rapid recovery toward the baseline level start 4-6 s after the onset of the rest epoch in OxyHb and HHb.<br><br>No significant decrease in the folding average HHb traces concurrent with the significant OxyHb increase observed during stimulations in the light channels outside the activated area.
**Duncan 1995-1 [82]**	INVOS 3100	?	Forehead operated side	P	22	N/A	N/A	Extra	ECA clamping	2 min before ICA clamp	Shunt group (n=10) ECA-clamp vs baseline (med (range)): 67 (58-74) to 68 (56-72) %, N.S.<br>No shunt group (n=12) ECA-clamp vs baseline (med (range)): 71 (63-83) to 70 (54-80) %, N.S.
**Duncan 1995-2 [82]**	INVOS 3100	?	Forehead operated side	P	22	N/A	N/A	Intra	ICA clamping	ICA clamp 2 min after ECA clamp	Shunt group (n=10) ICA-clamp vs ECA-clamp (med (range)): 68 (56-72) to 56 (46-65)%, p=0.05<br>No shunt group (n=12) ICA-clamp vs ECA-clamp (med (range)): 70 (54-80) to 64 (54-76)%, p=0.05
**Lam 1997-1 [83]**	NIRO-500	6	Forehead, avoiding temporal muscle and sagittal sinus	P	37	TCD	LDF	E	ECA clamping	ECA clamp 2 min before ICA clamp	Classified by authors as 'decreased', 'increased', 'no change'.<br>LDF: 23 decrease OxyHb with 23 decrease LDF; 8 no change OxyHHb with 8 no change LDF<br>21 increase HHb, with 21 decrease LDF; 10 no change HHb, with 8 no change LDF, 2 decrease LDF<br>TCD: 'Small reduction in CBFv (54 to 44 cm/s) in n=1 patient upon ECA clamping'
**Lam 1997-2 [83]**	NIRO-500	6	Forehead, avoiding temporal muscle and sagittal sinus	P	33	TCD	LDF	I	ICA clamping	ICA clamp 2 min after ECA clamp	LDF: For n=33 no change in LDF<br>TCD: 18 decrease OxyHb, 18 decrease CBFv; 14 no change OxyHb, 9 decease CBFv, 5 no change CBFv<br>18 increase HHb, 18 decrease CBFv; 14 no change HHb, 9 decrease CBFv, 5 no change CBFv
**Lam 1997-3 [83]**	NIRO-500	6	Forehead, avoiding temporal muscle and sagittal sinus	P	26	TCD	LDF	E	ECA unclamping	ECA unclamping before ICA unclamping, unclear period	LDF: 17 increase OxyHb, 14 increase LDF, 3 no change LDF; 9 no change OxyHb, 9 no change LDF<br>15 decrease HHb, 12 increase LDF, 3 no change LDF; 11 no change HHb, 1 increase

											LDF,10 no change LDF<br>TCD: n=2 increase CBFv (with increase LDF and OxyHb and decrease HHb)<br>LDF: n=2 increase LDF (with increase CBFv) and OxyHb)
**Lam 1997-4 [83]**	NIRO-500	6	Forehead, avoiding temporal muscle and sagittal sinus	P	36	TCD	LDF	I	ICA unclamping	ICA unclamping after ECA unclamping, unclear period	TCD: 17 increase OxyHb, 17 increase CBFv; 19 no change OxyHb, 16 increase CBFv, 3 no change CBFv<br>16 decrease HHb, 16 increase CBFv; 18 no change HHb, 11 increase CBFv, 7 no change CBFv. N.B.2 increase HHb, 2 increase CBFv
